# A Multi-Center Cohort-Based circRNA Diagnostic Model for Detection of Gastric Cancer

**DOI:** 10.1186/s12575-026-00326-4

**Published:** 2026-01-16

**Authors:** Xiaoyu Gu, Shuo Ma, Xun Gao, Chenyan Yuan, Wei Gao, Fengfeng Zhao, Yonghui Liu, Chen Zhang, Guoqiu Wu, Shuang Liu

**Affiliations:** 1 Center of Clinical Laboratory Medicine, Zhongda Hospital, Southeast University, Nanjing, Jiangsu China; 2 Department of Laboratory Medicine, Medical School of Southeast University, Nanjing, Jiangsu China

**Keywords:** Circular RNAs, Gastric cancer, Prediction model, Multicenter cohort

## Abstract

**Background:**

Gastric cancer (GC) remains one of the most detrimental diseases to human health. Owing to the subtle nature of early symptoms and the absence of robust and effective screening biomarkers, most patients are diagnosed at an advanced stage. Herein, our objective is to establish a non-invasive diagnostic strategy based on circular RNAs (circRNAs) to facilitate the detection of GC.

**Methods:**

We conducted a comprehensive genome-wide screening to identify key circRNAs, which were subsequently validated via RT-qPCR and translated into a plasma-based liquid biopsy analysis. The Chi-square test was applied to evaluate the relationship between circRNA expression and clinicopathological parameters. Receiver Operating Characteristic (ROC) curves were employed to evaluate the diagnostic efficacy of circRNAs in GC. A logistic regression model was established for the prediction of GC and validated in independent clinical cohorts.

**Results:**

In the discovery phase, we identified 2 circRNA candidates, hsa_circ_0001185 and hsa_circ_0005265, which were subsequently found to be significantly upregulated in the serum of GC patients through liquid biopsy analysis. The Chi-square test revealed that elevated expression of these circRNAs was significantly correlated with differentiation grade, lymph node metastasis, and TNM stage. ROC analysis demonstrated that hsa_circ_0001185 and hsa_circ_0005265 effectively discriminated GC patients from non-diseased controls, with AUC values of 0.909 and 0.853, respectively. The Diagnostic Model for GC (GC-DM) we developed exhibited an AUC of 0.915, which was subsequently validated in two independent cohorts.

**Conclusion:**

We developed a GC diagnostic model based on hsa_circ_0001185 and hsa_circ_0005265, demonstrating robust non-invasive diagnostic potential for the detection of GC patients.

**Supplementary Information:**

The online version contains supplementary material available at 10.1186/s12575-026-00326-4.

## Introduction

Gastric cancer (GC) is one of the most common malignant tumors worldwide. According to the GLOBOCAN 2022 report, it ranks fifth globally in both incidence and mortality rates [1]. Early GC often presents without typical symptoms, and most patients are diagnosed at advanced stages, leading to a poor prognosis. Advanced GC, despite radical resection, still carries a high risk of metastasis and recurrence [2, 3]. Hence, early screening for GC is crucial for improving patient prognosis and enhancing survival rates. Currently, GC diagnosis predominantly depends on gastroscopy and pathological biopsy, which require advanced equipment and skilled operators. Moreover, minimally invasive procedures often cause discomfort to patients [4]. In addition, clinically utilized plasma-based biomarkers for GC diagnosis include carcinoembryonic antigen (CEA), carbohydrate antigens CA199 and CA724, as well as pepsinogen and alpha-fetoprotein [5]. These indicators lack sufficient sensitivity (SEN) and specificity (SPE) to serve as biomarkers for the detection of GC, leading to a delay in treatment for many GC patients. Hence, it is critical to identify and develop high-sensitivity, high-specificity non-invasive biomarkers for GC screening and therapeutic evaluation.

Circular RNAs (circRNAs) are single-stranded RNAs formed by back-splicing of precursor mRNA, characterized by a covalently closed structure without a 5′ cap and 3′ poly(A) tail. CircRNAs are more resistant to ribonucleases, conferring enhanced stability compared to linear mRNAs [6, 7]. In recent years, circRNAs have been demonstrated to play a pivotal role in the pathogenesis of several solid cancers, including GC [8–12]. Studies have shown that circRNAs can be detected in various tissue-specific bodily fluids (including plasma, urine, saliva, and feces) with stable expression. Additionally, circRNAs are covalently closed structures that exhibit a prolonged half-life and are cell-type specific. These characteristics suggest that circRNAs may hold untapped potential as biomarkers in liquid biopsy [13, 14]. Nevertheless, research on circRNAs is still in its infancy, and the development prospects of circRNA-based biomarkers remain uncertain. Further investigation of GC-related circRNAs will provide valuable insights for screening and identifying novel diagnostic and therapeutic strategies for GC.

In this study, we systematically and comprehensively identified more sensitive and specific diagnostic biomarkers for GC by comparing circRNA expression profiles in serum samples from GC patients and matched controls. These efforts enabled us to screen 2 key circRNAs, hsa_circ_0001185 and hsa_circ_0005265, which were significantly upregulated in the serum of GC patients. Research indicates that hsa_circ_0001185 and hsa_circ_0005265 demonstrate robust diagnostic accuracy in GC, with their performance further enhanced when combined with currently utilized classic tumor markers. We were able to significantly differentiate potential patients who may develop GC by constructing a diagnostic model for GC (GC-DM) based on these circRNAs. Thus, our research provides novel avenues for the detection of GC.

## Methods

### Study Design and Patient Cohorts

This study was approved by the Clinical Research Ethics Committee of Affiliated Hospital of Nantong University (Ethical Review Report No: 2018-L055), and informed written consent was obtained from all participants before the clinical trial. All patients were pathologically diagnosed and had not received neoadjuvant radiotherapy, chemotherapy, or targeted therapy before surgery. This study encompasses three critical phases: a circRNA-based biomarker identification phase, a plasma-based validation phase, a multi-center cohort study-based model construction and performance evaluation phase. At the initial stage of biomarker identification, we downloaded and reanalyzed the expression profile data from the Gene Expression Omnibus (GEO) database (GSE152309), accessible at https://www.ncbi.nlm.nih.gov/geo/query/acc.cgi?acc=GSE152309. In the plasma-based validation phase, the expression levels of candidate circRNAs were assessed via RT-qPCR in serum samples from GC patients, gastritis patients, and matched healthy controls. This phase involved 152 preoperative GC patients, 160 gastritis patients, and 148 healthy controls enrolled at Zhongda Hospital, Medical School of Southeast University, between 2022 and 2024. Finally, in the multi-center cohort study-based model construction and performance evaluation phase, patient samples from Zhongda Hospital were randomly assigned in a 1:1 ratio to the training and internal validation groups. In addition, GC serum samples from two independent centers were also collected between 2022 and 2024. The study is divided into four cohorts: (1) Training cohort: 76 GC patients, 80 gastritis patients, and 74 healthy controls from Zhongda Hospital. (2) Internal validation cohort: 76 GC patients, 80 gastritis patients, and 74 healthy controls from Zhongda Hospital. (3) Independent external validation cohort 1: 44 GC patients and 42 controls from China-Japan Friendship Hospital. (4) Independent external validation cohort 2: 42 GC patients and 40 healthy controls from Affiliated Hospital of Nantong University.

### Construction and Validation of the Diagnostic Model for GC (GC-DM)

Leveraging the expression levels of hsa_circ_0001185 and hsa_circ_0005265 in GC serum samples, we utilized these circRNAs’ expression levels from the training cohort of Zhongda Hospital as independent variables, with GC outcomes as the dependent variable. Risk scores for each case were derived from the coefficients generated by logistic regression analysis to construct a robust diagnostic model for GC (GC-DM). GC-DM = −4.962 + 2.989 × Exp (hsa_circ_0001185) + 6.651 × Exp (hsa_circ_0005265). The diagnostic performance of the model was validated through ROC curve analysis, yielding key metrics such as sensitivity and specificity to determine the optimal cut-off value. To validate the stability of the GC-DM model, we utilized the internal validation cohort from Zhongda Hospital, and additionally collected patient cohorts from China-Japan Friendship Hospital and Affiliated Hospital of Nantong University. Using the cutoff value established in the model, we applied the same diagnostic formula and statistical analyses to further validate the performance of GC-DM.

### Serum Sample Processing, RNA Extraction, and Reverse Transcription Quantitative Polymerase Chain Reaction (RT-qPCR)

To ensure RNA stability and minimize pre-analytical variability, a standardized protocol was implemented across all centers. Peripheral blood was collected in serum separation tubes, allowed to clot at room temperature for 30–60 min, and centrifuged at 1,600 × g for 10 min at 4 °C. The harvested serum underwent a second centrifugation at 12,000 × g for 10 min at 4 °C to remove residual cells and debris before being aliquoted and stored at −80 °C. Samples with visible hemolysis, lipemia, or icterus were excluded. All biomarker analyses (circRNAs and conventional tumor markers) for a given individual were performed on the same serum aliquot. RNA was extracted from 250 µL of serum using the Serum Total RNA Extraction Kit (BioTeke Corporation). RNA concentration and purity (A260/A280) were measured, and integrity was verified by agarose gel electrophoresis. RT-qPCR analysis was performed using the Applied Biosystems™ 7500 Real-Time PCR System (Thermo Fisher Scientific, Massachusetts, USA), with amplification of a 20 µL reaction using Taq Pro Universal SYBR qPCR Master Mix. The reference gene used was 18S rRNA. Table 1 lists the specific primers for circRNAs (Sangon Biotech, Shanghai, China).

**Table 1:** Primer sequences used in this study. CircRNAs: circular RNAs

	Primer sequences (5ʹ−3ʹ)	
circRNAs	Forward	Reverse
hsa_circ_0007919	TTTGAGATCGAGCTGGAGGG	TTTCCCTGCTTCCACCTGG
hsa_circ_0002874	CAAAGCAGCAGGAGTTTGGA	CGGATGGCAGGAATGTGATG
hsa_circ_0001681	GGTGGCATCTGTGAACTGTC	GCTCCTCCTGTAAAACCCCT
hsa_circ_0001185	AGAAGAGGTCAAAGGCCCTT	TGTTGCTTAAATGCCCGACT
hsa_circ_0005265	TAACCAGTTCACGGATGCCA	GCTCTTTGAACATGTGTCCTGA
18s rRNA	CGGCTACCACATCCAAGGAA	GCTGGAATTACCGCGGCT

### Agarose Gel Electrophoresis

A 3% agarose gel was prepared in TAE buffer by heating until fully dissolved, then cast at room temperature. PCR products were mixed with loading buffer (5:1, v/v), loaded onto the gel, and electrophoresed at 110 V for 30–60 min. Bands were visualized under UV illumination and photographed.

### RNase R and Actinomycin D Assays

5 µL of total RNA was treated with RNase R (Geneseed, China), followed by inactivation of RNase R at 85 °C for 10 min. The treated RNA was then reverse transcribed into cDNA for subsequent PCR validation. Cells were treated with actinomycin D (2.5 µg/mL) for 0, 4, 8, 12, 24, and 48 h, after which total RNA was extracted for PCR analysis.

### Cell Culture and Transfection

GC cells (HGC-27, AGS) and gastric epithelial cells (GES-1) were provided by the Chinese Academy of Sciences (Shanghai, China). All cells were cultured in RPMI 1640 medium (Corning, Manassas, VA, USA) supplemented with 10% fetal bovine serum (Gibco, Waltham, MA, USA) and 1% penicillin-streptomycin (HyClone, Logan, UT, USA).

### Statistical Analysis

All statistical analyses were conducted using GraphPad Prism 9.5 and SPSS version 26.0. Variables were expressed as mean ± standard deviation, median (interquartile range), or percentages, based on their distribution. The “limma” package was used for DEC analysis between GC and matched control groups. “ggplot2” and “pheatmap” were used to generate volcano plots and heatmaps. The Kolmogorov-Smirnov test was initially applied to determine the normality of data distribution. For variables with a normal distribution, homogeneity of variance was tested. When normality and homogeneity of variance were confirmed, an independent samples t-test was applied. Otherwise, the Mann-Whitney U test was used. A p-value of < 0.05 was considered statistically significant. ROC curve analysis was conducted to assess the accuracy of circRNAs in distinguishing between different groups. The optimal cutoff value for the ROC curve was determined using the Youden index in the “pROC” package.

## Results

### Identification of circRNA Expression Profiles

**Fig. 1:**
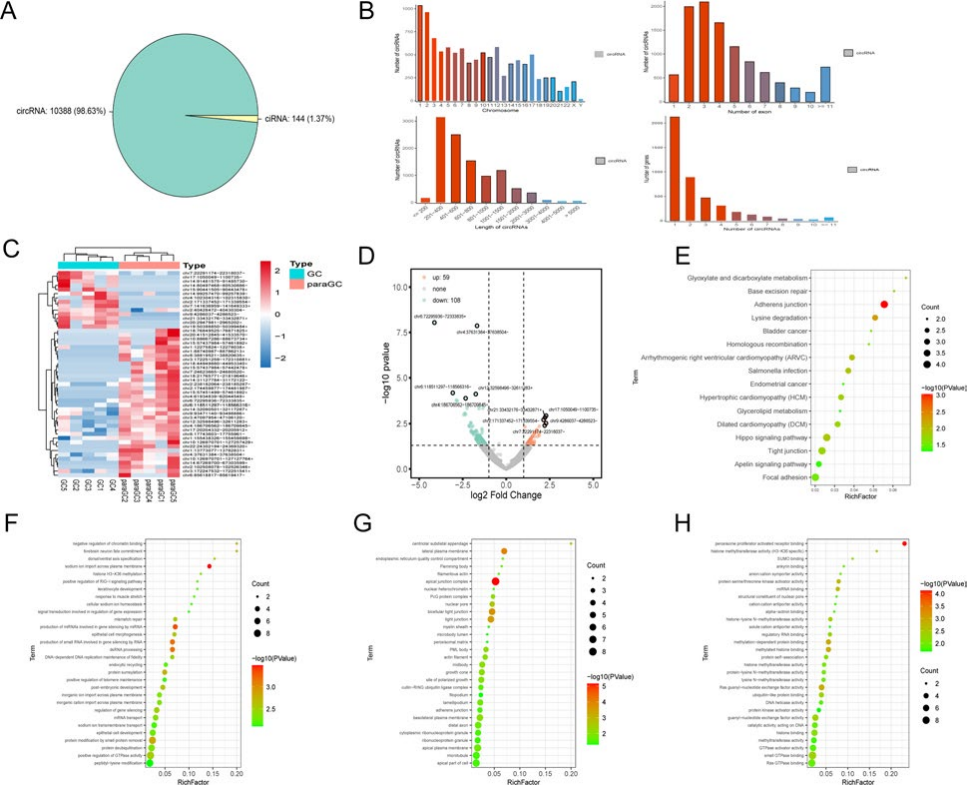
Expression profile of circRNAs in GC and screening of key circRNAs.**A** Types of circRNAs. **B** Chromosomal locations, exon compositions, lengths, and number of circRNAs generated from individual genes after sequencing. **C** Heatmap of differential circRNAs between GC patients and matched adjacent normal mucosa. **D** Volcano plot represents the significantly up- (59) and down-regulated (108) circRNAs derived from the GSE152309 dataset. **E** KEGG enrichment analysis of differential circRNAs. **F-H** GO functional enrichment analysis of differential circRNAs. GC, Gastric cancer

To investigate the expression profiles of circRNAs in GC, we performed a comprehensive analysis of next-generation sequencing (NGS) data from five GC tissues and their matched paracancerous tissues, sourced from the Gene Expression Omnibus (GEO) database (GSE152309). A total of 10,532 circRNAs were identified. Sequencing results revealed that these circRNAs are distributed across all chromosomes, with a predominant concentration on chromosomes 1 and 2. The majority of circRNAs are generated by the circularization of 2 to 4 exons, with lengths ranging from 201 to 600 base pairs. Additionally, most genes give rise to only 1 or 2 circRNAs, highlighting the specificity of circRNA production (Fig. 1A and B). We then screened for differentially expressed circRNAs between the two groups. Applying a threshold of log_2_|FC| ≥ 2 and *P* ≤ 0.05, we identified 167 circRNAs with significantly altered expression, including 59 upregulated and 108 downregulated circRNAs (Fig. 1C and D). Kyoto Encyclopedia of Genes and Genomes (KEGG) pathway analysis showed that these differentially expressed circRNAs primarily exert their roles in GC through pathways such as glyoxylate and dicarboxylate metabolism, base excision repair, and adherens junctions (Fig. 1E). Furthermore, Gene Ontology (GO) analysis showed that the identified circRNAs are primarily associated with processes such as negative regulation of chromatin binding, centriolar subdistal appendage, peroxisome proliferator-activated receptor binding, and histone methyltransferase activity (H3-K36 specific) (Fig. 1F-H). These findings highlight the significant alterations of the circRNA expression profile in GC, suggesting the potential role of these circRNAs in regulating the GC microenvironment through various mechanisms.

### Screening and Identification of hsa_circ_0001185 and hsa_circ_0005265

**Fig. 2:**
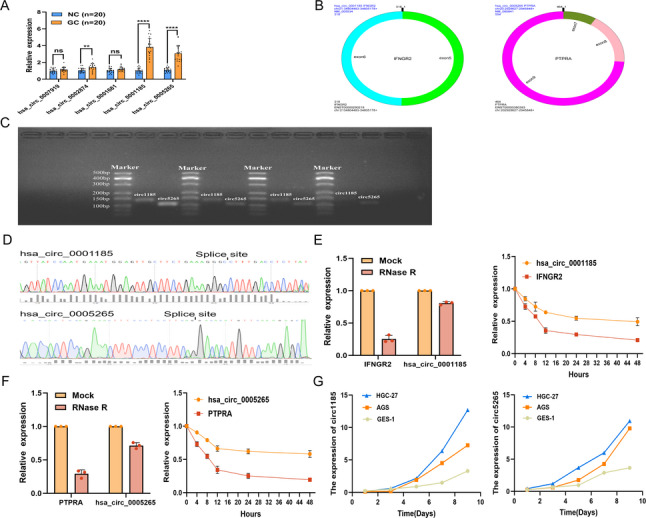
Screening and identification of hsa_circ_0001185 and hsa_circ_0005265.**A** The expression levels of the five selected circRNAs in the serum of GC patients (*n* = 20) compared to the matched HC group (*n* = 20). **B** Basic information of hsa_circ_0001185 and hsa_circ_0005265. **C** Agarose gel electrophoresis results of hsa_circ_0001185 and hsa_circ_0005265. **D** Sanger sequencing detects the cyclization sites. **E-F** Expression of circRNAs and their host genes after treatment with RNase R enzyme or actinomycin D. **G** Secretion of circRNAs in GC cells and gastric epithelial cells. **P < 0.01, ***P < 0.001, ****P < 0.0001. NC, Normal control; GC, Gastric cancer

To develop a blood-based, non-invasive diagnostic approach for GC, we prioritized the 59 circRNAs upregulated by sequencing. The top five circRNAs were selected for further analysis, and their expression levels were measured in the serum of 20 GC patients and matched controls (Table 1). Notably, hsa_circ_0001185 and hsa_circ_0005265 exhibited marked upregulation in the serum of GC patients (Fig. 2A), consistent with our sequencing data. Moreover, these two circRNAs have not been previously reported in GC, prompting us to further investigate their potential as GC biomarkers. Using the circPrimer software, we found that hsa_circ_0001185 and hsa_circ_0005265 are derived from the IFNGR2 and PTPRA genes, respectively. hsa_circ_0001185 is primarily formed by the circularization of the fifth and sixth exons of the IFNGR2 gene, with a length of 318 bp, while hsa_circ_0005265 is mainly derived from the seventh, eighth, and ninth exons of the PTPRA gene, with a length of 554 bp (Fig. 2B). Agarose gel electrophoresis and Sanger sequencing confirmed the specificity of the primers for these two circRNAs. The electrophoresis bands were observed at 140 bp and 88 bp, respectively, which corresponded to the expected sizes of the primer amplification products (Fig. 2C and D). We treated AGS cells with RNase R and actinomycin D, and found that both circRNAs exhibited higher stability compared to their corresponding linear parent genes (Fig. 2E and F). To investigate the secretion dynamics of these circRNAs, we cultured GES-1, HGC-27, and AGS cells for 1, 3, 5, 7, and 9 days, and measured the expression levels of hsa_circ_0001185 and hsa_circ_0005265 in cell culture supernatants. Both circRNAs increased over time, with a more pronounced rise in GC cell lines (Fig. 2G), supporting their potential as serum biomarkers for GC.

### Expression Characteristics and Clinical Value of hsa_circ_0001185 and hsa_circ_0005265 in GC Serum

**Fig. 3:**
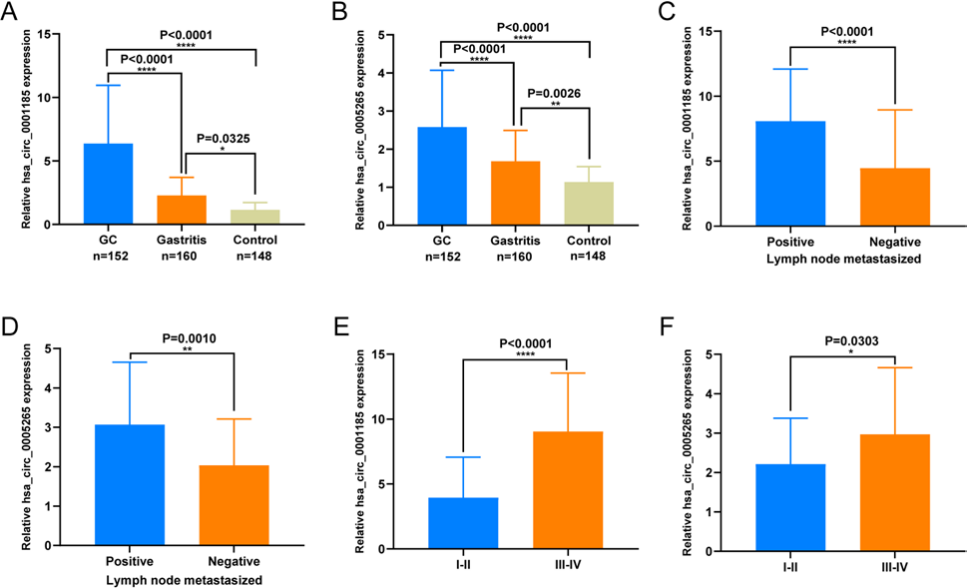
Expression characteristics and clinical value of hsa_circ_0001185 and hsa_circ_0005265 in GC serum. **A **and** B** The expression levels of hsa_circ_0001185 and hsa_circ_0005265 in the serum of GC patients (*n* = 152), gastritis patients (*n* = 160), and healthy donors (*n* = 148). **C **and** D** The expression levels of hsa_circ_0001185 and hsa_circ_0005265 in GC patients with or without lymph node metastasis. **E **and** F** The expression levels of hsa_circ_0001185 and hsa_circ_0005265 in GC patients with different tumor node metastasis stages. *P < 0.05, **P < 0.01, ****P < 0.0001. GC, Gastric cancer

We gathered serum samples from 152 GC patients, 160 gastritis patients, and 148 healthy donors to evaluate the expression patterns and clinical significance of hsa_circ_0001185 and hsa_circ_0005265. The results showed that serum levels of both hsa_circ_0001185 and hsa_circ_0005265 were significantly higher in GC patients than in gastritis patients and healthy donors. Notably, gastritis patients also exhibited markedly elevated levels compared with matched healthy controls, yielding a stepwise pattern across groups (healthy controls < gastritis < GC), with GC levels remaining significantly higher than those in gastritis (Fig. 3A and B). To investigate the potential association between the two circRNAs and clinical characteristics, we stratified the GC cohort into high- and low-expression groups according to the median expression levels of hsa_circ_0001185 and hsa_circ_0005265. The χ² test was employed to evaluate the correlation between the expression levels of these circRNAs and clinicopathological parameters. We found that the expression level of hsa_circ_0001185 was significantly associated with the differentiation degree, lymph node metastasis, TNM stage, histological type, and nerve/vascular invasion of GC patients (Table 2). Similarly, the expression level of hsa_circ_0005265 was correlated with TNM stage, nerve/vascular invasion, depth of infiltration, and CK status (Table 3). We further analyzed the differences in the expression levels of hsa_circ_0001185 and hsa_circ_0005265 among the subgroups significantly associated with clinicopathological parameters. The results showed that the expression levels of hsa_circ_0001185 and hsa_circ_0005265 were higher in the lymph node metastasis group, the high malignancy grade (III-IV) group, the deeper infiltration group, and the nerve/vascular invasion group (Fig. 3C-F and Supplementary Fig. 1A-F). This underscores the significant association between the expression of these circRNAs and the malignancy of GC, suggesting their potential critical role in the development and progression of the disease.

**Table 2:** Clinicopathological analysis of hsa_circ_0001185 in GC

Parameter		No. of patients	hsa_circ_0001185 (high)	hsa_circ_0001185 (low)	*P*-value
Sex	Male	122	64	58	0.2214
	Female	30	12	18	
Age(years)	≤ 60	42	20	22	0.7168
	>60	110	56	54	
Grade	Well-moderate	66	18	48	< 0.0001^****^
	Poor-undifferentiation	86	58	28	
Pathologic type	Adenocarcinoma	114	62	52	0.001^***^
	Adenosquamous carcinoma	8	0	8	
	Signet-ring cell carcinoma	16	10	6	
	Mucinous adenocarcinoma	6	4	2	
	Undifferentiated carcinoma	8	0	8	
Lymph node status	Positive	90	60	30	< 0.0001^****^
	Negative	62	16	46	
TNM stage	Ⅰ-Ⅱ	70	22	48	< 0.0001^****^
	Ⅲ-Ⅳ	82	54	28	
Nerve/vascular invasion	Positive	86	50	36	0.0220^*^
	Negative	66	26	40	
Depth of infiltration	Negative	38	16	22	0.2611
	Submucosa and above	114	60	54	
Ki67	Positive	118	64	54	0.0516
	Negative	34	12	22	
CK	Positive	50	28	22	0.3003
	Negative	102	48	54	

*circ* circular, *GC* Gastric cancer, *TNM* Tumor node metastasis

*P < 0.05, **P < 0.01, ***P < 0.001, ****P < 0.0001

**Table 3:** Clinicopathological analysis of hsa_circ_0005265 in GC

Parameter		No. of patients	hsa_circ_0005265(high)	hsa_circ_0005265(low)	*P*-value
Sex	Male	122	60	62	0.6836
	Female	30	16	14	
Age(years)	≤ 60	42	20	22	0.7168
	>60	110	56	54	
Grade	Well-moderate	66	32	34	0.7434
	Poor-undifferentiation	86	44	42	
Pathologic type	Adenocarcinoma	114	58	56	0.4479
	Adenosquamous carcinoma	8	4	4	
	Signet-ring cell carcinoma	16	10	6	
	Mucinous adenocarcinoma	6	2	4	
	Undifferentiated carcinoma	8	2	6	
Lymph node status	Positive	90	50	40	0.0988
	Negative	62	26	36	
TNM stage	Ⅰ-Ⅱ	70	22	48	< 0.0001^****^
	Ⅲ-Ⅳ	82	54	28	
Nerve/vascular invasion	Positive	86	52	34	0.0032^**^
	Negative	66	24	42	
Depth of infiltration	Negative	38	12	26	0.0087^**^
	Submucosa and above	114	64	50	
Ki67	Positive	118	62	56	0.2429
	Negative	34	14	20	
CK	Positive	50	18	32	0.0157^*^
	Negative	102	58	44	

*circ* circular, *GC* Gastric cancer, *TNM* Tumor node metastasis

*P < 0.05, **P < 0.01, ***P < 0.001, ****P < 0.0001

### The Liquid Biopsy-Based circRNA Molecules Robustly Identify Patients with GC

**Fig. 4:**
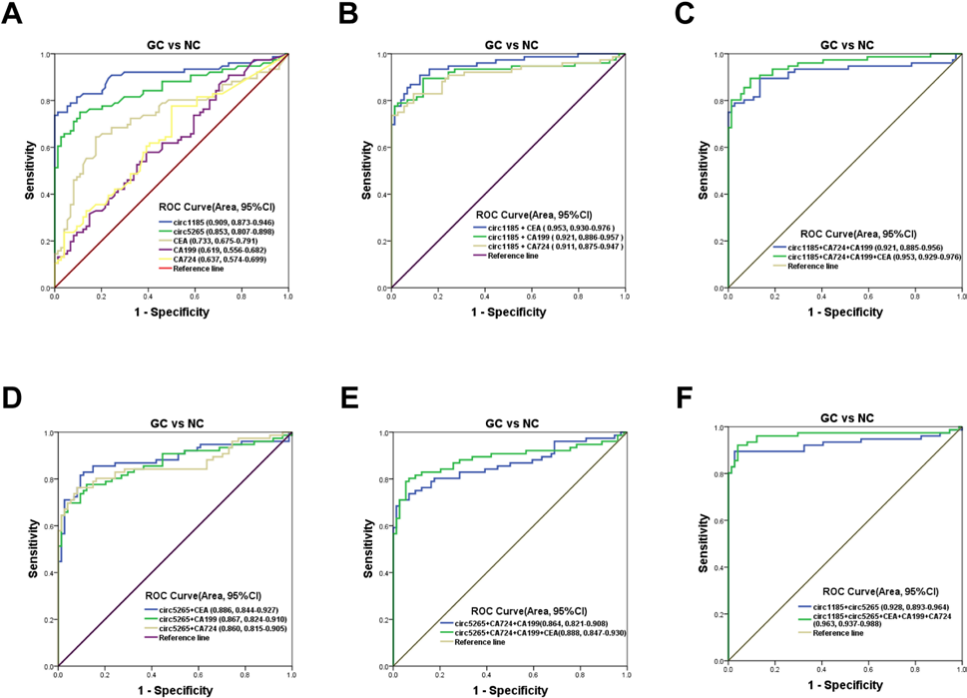
Diagnostic value of serum hsa_circ_0001185 and hsa_circ_0005265 in GC. **A** ROC curve analysis of hsa_circ_0001185, hsa_circ_0005265, CEA, CA199 and CA724 in independent diagnosis of GC patients and healthy donors. **B-F** Diagnostic efficacy evaluation of combined hsa_circ_0001185 and hsa_circ_0005265 in GC patients and matched controls. GC, Gastric cancer; ROC, Receiver Operating Characteristic curve; AUC, Area Under the Curve; CI, Confidence Interval

In order to develop a clinically feasible diagnostic method for GC with high sensitivity, we performed ROC analysis to determine the diagnostic efficacy of hsa_circ_0001185, hsa_circ_0005265, and traditional GC tumor markers (CEA, CA199, and CA724) in GC. In the single marker ROC diagnostic analysis, the AUC values of hsa_circ_0001185 and hsa_circ_0005265 for diagnosing GC were 0.909 and 0.853, significantly higher than those of CEA (AUC = 0.733), CA199 (AUC = 0.619), and CA724 (AUC = 0.637) (Fig. 4A). At this point, the SEN and SPE of hsa_circ_0001185 in diagnosing GC were 0.74 and 0.89, respectively, while hsa_circ_0005265 exhibited a SEN of 0.75 and SPE of 0.88. Both were significantly higher than CEA (SEN = 0.64, SPE = 0.82), CA199 (SEN = 0.57, SPE = 0.69), and CA724 (SEN = 0.70, SPE = 0.64) (Table 4). Subsequently, we performed a combined ROC curve analysis of several biomarkers. The results showed that the combination of hsa_circ_0001185 with CEA, CA199, and CA724 for diagnosis yielded an AUC of 0.953 (Fig. 4B-C), with both SEN and SPE at 0.89 (Table 4). Likewise, when hsa_circ_0005265 was combined with CEA, CA199, and CA724, the AUC reached 0.888 (Fig. 4D-E), with SEN and SPE achieving 0.80 and 0.93, respectively (Table 4). Additionally, following a combined analysis of hsa_circ_0001185 and hsa_circ_0005265, we observed that the combined AUC for hsa_circ_0001185 and hsa_circ_0005265 reached 0.928, and SEN and SPE were 0.89 and 0.97, respectively. Ultimately, after combining all the above markers, the AUC of the ROC curve yielded 0.963 (Fig. 4F), with SEN and SPE improving to 0.92 and 0.96, respectively (Table 4).

**Table 4:** The diagnostic performance of circ1185, circ5265, CEA, CA199, and CA724 in differentiating GC patients from healthy donors

	SEN	SPE	ACCU	PPV	NPV
circ1185	0.74 (112/152)	0.89 (132/148)	0.81 (244/300)	0.88 (112/128)	0.77 (132/172)
circ5265	0.75 (114/152)	0.88 (130/148)	0.81 (244/300)	0.86 (114/132)	0.77 (130/168)
CEA	0.64 (98/152)	0.82 (122/148)	0.73 (220/300)	0.79 (98/124)	0.69 (122/176)
CA199	0.57 (87/152)	0.69 (102/148)	0.63 (189/300)	0.65 (87/133)	0.61 (102/167)
CA724	0.70 (106/152)	0.64 (94/148)	0.67 (200/300)	0.66 (106/160)	0.68 (94/138)
circ1185 + CEA	0.87 (132/152)	0.92 (136/148)	0.90 (272/300)	0.92 (136/148)	0.93 (136/146)
circ1185 + CA199	0.78 (118/152)	0.86 (128/148)	0.82 (246/300)	0.86 (118/138)	0.79 (128/162)
circ1185 + CA724	0.74 (113/152)	0.88 (130/148)	0.81 (243/300)	0.86 (113/131)	0.77 (130/169)
circ1185 + CA724 + CA199	0.78 (119/152)	0.84 (125/148)	0.81 (244/300)	0.84 (119/142)	0.79 (125/158)
circ1185 + CA724 + CA199 + CEA	0.89 (136/152)	0.89 (132/148)	0.89 (268/300)	0.89 (136/152)	0.89 (132/148)
circ5265 + CEA	0.82 (124/152)	0.89 (132/148)	0.85 (256/300)	0.89 (124/140)	0.83 (132/160)
circ5265 + CA199	0.70 (106/152)	0.87 (129/148)	0.78 (235/300)	0.85 (106/125)	0.74 (129/175)
circ5265 + CA724	0.76 (115/152)	0.90 (133/148)	0.83 (248/300)	0.88 (115/130)	0.78 (133/170)
circ5265 + CA724 + CA199	0.71 (108/152)	0.89 (132/148)	0.80 (240/300)	0.87 (108/124)	0.75 (132/176)
circ5265 + CA724 + CA199 + CEA	0.80 (122/152)	0.93 (138/148)	0.87 (260/300)	0.92 (122/132)	0.82 (138/168)
circ1185 + circ5265	0.89 (136/152)	0.97 (144/148)	0.93 (280/300)	0.97 (136/140)	0.90 (144/160)
circ1185 + circ5265 + CEA + CA199 + CA724	0.92 (140/152)	0.96 (142/148)	0.94 (282/300)	0.96 (140/146)	0.92 (142/154)

*GC-DM* Diagnostic model for GC, *SEN* Sensitivity, *SPE* Specificity, *ACCU* Overall accuracy, *NPV* Negative predictive value, *PPV* Positive predictive value

Most GC cases originate from chronic gastritis, and early-stage GC shares similar clinical manifestations with gastritis [15]. The accurate differentiation between GC and gastritis is essential for prompt and effective treatment, ultimately enhancing patient prognosis. To assess the discriminatory potential of these two circular RNAs in differentiating GC patients from gastritis cases, ROC curve analysis was performed. The findings showed that the diagnostic AUC for hsa_circ_0001185 was 0.784, with a SEN of 0.59 and SPE of 0.93. Similarly, circ5265 achieved an AUC of 0.720, with both SEN and SPE reaching 0.70, significantly higher than CEA (AUC = 0.658, SEN = 0.45, SPE = 0.88), CA199 (AUC = 0.605, SEN = 0.47, SPE = 0.75), and CA724 (AUC = 0.583, SEN = 0.38, SPE = 0.83) (Supplementary Fig. 2A and Supplementary Table 1). When all five biomarkers were combined, the AUC increased to the highest value of 0.864, with SEN and SPE of 0.80 and 0.84, respectively (Supplementary Fig. 2B-F and Supplementary Table 1). Furthermore, ROC analysis indicated that these two circRNAs exhibit a strong capability to distinguish gastritis patients from non-diseased controls. The AUC for diagnosing gastritis using hsa_circ_0001185 and hsa_circ_0005265 was 0.746 and 0.722, respectively, with the combined AUC reaching 0.862 (Supplementary Fig. 3A-F). The combined biomarkers exhibited a SEN of 0.68 and SPE of 0.89 (Supplementary Table 2). Overall, our results underscored that the combined use of hsa_circ_0001185 and hsa_circ_0005265 holds substantial promise as diagnostic biomarkers for GC, exhibiting high sensitivity and specificity across GC patients.

### Development of a Diagnostic Model for GC (GC-DM) using circRNAs and validation in an internal independent cohort

**Fig. 5:**
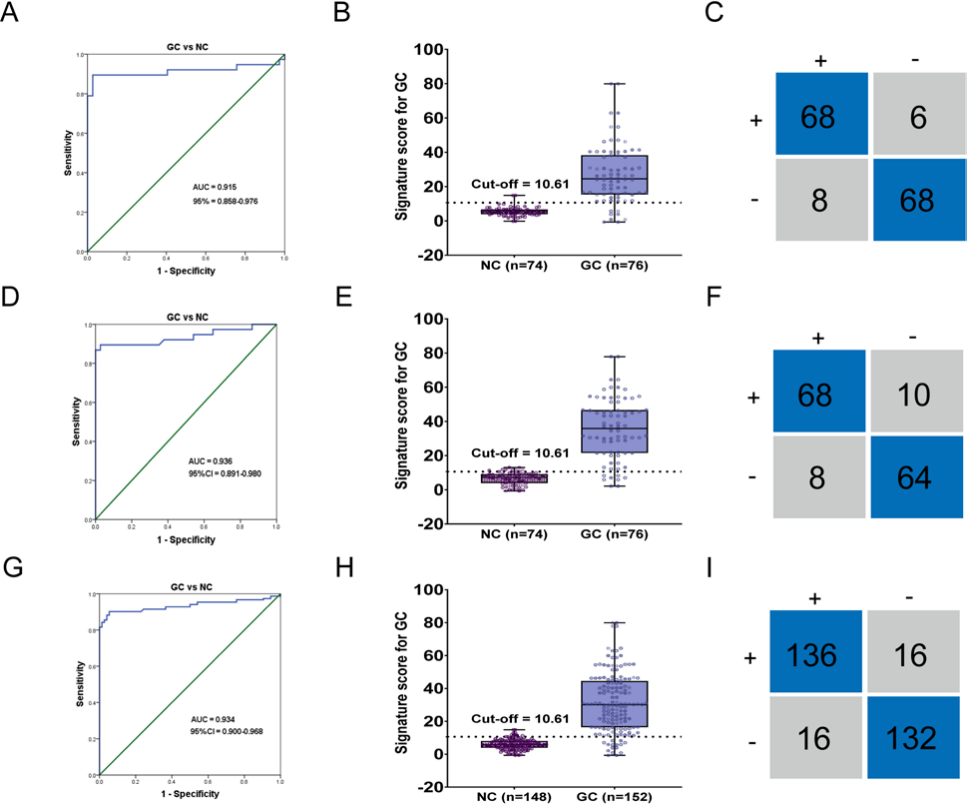
Construction and validation of the diagnostic model for GC.** A** The ROC curve for distinguishing GC patients in the training cohort using the model. **B** The optimal cut-off value set by the model. **C** Matrix diagram represents sensitivity and specificity of the model in the training cohort. **D-E** The ROC curve and cut-off value for distinguishing GC patients in the internal validation cohort using the model. **F** Matrix diagram represents sensitivity and specificity of the model in the internal validation cohort. **G-H** The ROC curve and cut-off value for distinguishing GC patients after combining the training cohort and internal validation cohort using the model. **I** The matrix diagram represents sensitivity and specificity of the model after combining the training cohort and internal validation cohort. GC, Gastric cancer; ROC, Receiver Operating Characteristic curve; AUC, Area Under the Curve

To enhance the diagnostic accuracy and clinical feasibility for GC, we divided Cohort 1 (patients from Zhongda Hospital) into a training cohort and an internal validation cohort in a 1:1 ratio; each group consisted of 76 GC patients, 80 gastric inflammation patients, and 74 healthy controls. Subsequently, a logistic regression model was constructed based on coefficients derived from the training cohort, with the formula: GC-DM = −4.962 + 2.989 × Exp (hsa_circ_0001185) + 6.651 × Exp (hsa_circ_0005265). The diagnostic model for GC (GC-DM) in the training cohort demonstrated an AUC of 0.915, with sensitivity and specificity of 0.89 and 0.92, respectively (Fig. 5A-C and Table 5). To achieve the maximum clinical diagnostic efficiency as well as optimal sensitivity and specificity, we set the cut-off value of the model at 10.61, with all subsequent cohort analyses conducted based on this threshold. We then validated the model’s accuracy via the internal validation cohort. The validation set demonstrated an AUC of 0.936, with sensitivity and specificity values of 0.89 and 0.86, respectively (Fig. 5D-F and Table 5). After combining the two cohorts, the overall AUC reached 0.934, with a sensitivity of 0.89 and specificity of 0.89, indicating strong diagnostic performance of the model (Fig. 5G-I and Table 5).

**Table 5:** The diagnostic performance of GC-DM in multiple independent clinical cohorts

	**SEN**	**SPE**	**ACCU**	**PPV**	**NPV**
Training cohort	0.89 (68/76)	0.92 (68/74)	0.91 (136/150)	0.92 (68/74)	0.89 (68/76)
Internal validation cohort	0.89 (68/76)	0.86 (64/74)	0.88 (132/150)	0.87 (68/78)	0.89 (64/72)
Combined cohort	0.89 (136/152)	0.89 (132/148)	0.89 (268/300)	0.89 (136/152)	0.89 (132/148)
External validation cohort 1	0.75 (33/44)	0.83 (35/42)	0.79 (68/86)	0.83 (33/40)	0.76 (35/46)
External validation cohort 2	0.81 (34/42)	0.85 (34/40)	0.83 (68/82)	0.85 (34/40)	0.81 (34/42)

### CircRNA-based Diagnostic Model Was Independently Validated in Two Separate Cohorts of GC Patients

**Fig. 6:**
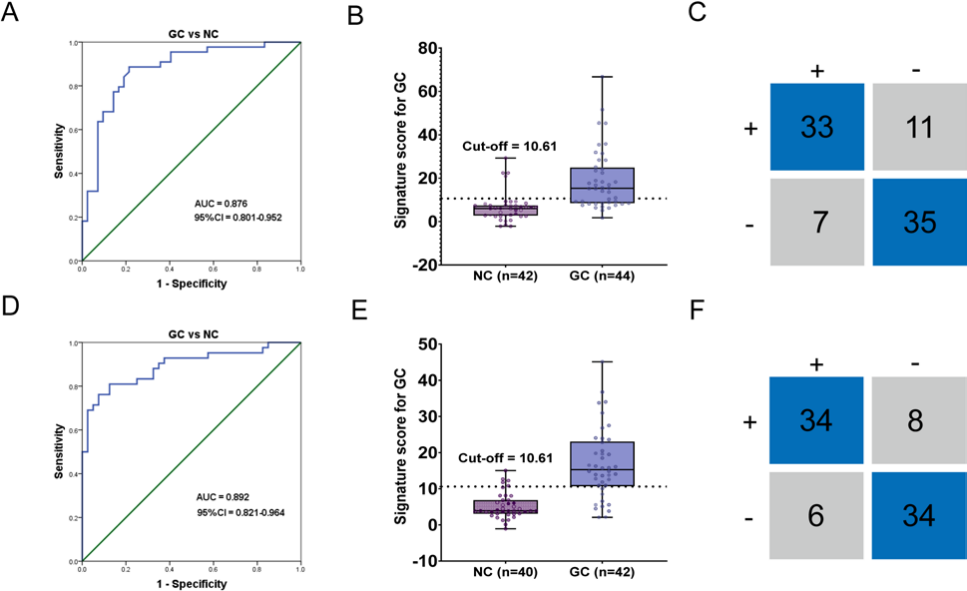
Validation of the diagnostic model in two independent cohorts of patients with GC.** A - B** The ROC curve and cut-off value for distinguishing GC patients in validation cohort 1 based on the model. **C** The matrix diagram represents sensitivity and specificity of the model in validation cohort 1. **D - E** The ROC curve and cutoff value for distinguishing GC patients in validation cohort 2 based on the model. **F** The matrix diagram represents sensitivity and specificity of the model in validation cohort 2. GC, Gastric cancer; ROC, Receiver Operating Characteristic curve; AUC, Area Under the Curve

Following the development of the GC-DM based on hsa_circ_0001185 and hsa_circ_0005265 in the training cohort, we collected serum specimens from two independent cohorts as external validation cohorts to further assess the diagnostic performance of the model. The clinical characteristics of these validation cohorts revealed that the expression levels of these two circRNAs were significantly elevated compared to the matched control groups, with both hsa_circ_0001185 and hsa_circ_0005265 showing notably higher expression in the lymph node metastasis group and the poorly differentiated GC group (Supplementary Fig. 4–5 and Supplementary Tables 3–6). ROC analysis showed that when the five biomarkers were analyzed in combination, the AUC value reached 0.916 in the independent validation cohort 1, with SEN and SPE of 0.82 and 0.95, respectively, while in the independent validation cohort 2, the AUC value reached 0.937, with SEN and SPE of 0.74 and 0.95, respectively (Supplementary Fig. 6–7 and Supplementary Tables 7–8). Subsequently, we analyzed these two validation cohorts based on the logistic regression model and found that hsa_circ_0001185 and hsa_circ_0005265 performed excellently in distinguishing GC patients from controls. GC-DM demonstrated an AUC value of 0.876 for validation cohort 1, with sensitivity and specificity of 0.75 and 0.83, respectively (Fig. 6A-C and Table 5). For validation cohort 2, the AUC value was 0.892, with SEN and SPE of 0.81 and 0.85, respectively (Fig. 6D-F and Table 5). Overall, our results highlighted that the cut-off value of 10.61 for the model exhibits strong robustness in distinguishing GC patients, further confirming its potential as a predictive tool for GC.

## Discussion

GC remains a major public health challenge, imposing substantial burdens on healthcare systems and society. According to the Global Cancer Statistics 2022, there were approximately 968,000 new cases and 660,000 deaths attributed to GC worldwide [1]. Despite recent therapeutic advances, GC remains challenging due to low early-detection rates and wide disparities in 5-year survival [16–18]. These challenges are largely attributed to nonspecific early symptoms and the lack of reliable biomarkers, leading to predominantly late-stage diagnoses when treatment options are severely limited. Histopathology remains the gold standard for GC diagnosis [19], which requires endoscopic biopsy [20, 21]. However, its adaptation to GC screening has been challenging due to the invasive nature, high cost, and patient discomfort [22]. Accordingly, non-invasive, highly specific molecular biomarkers are needed to enable earlier detection, timely intervention, and to improve patient outcomes.

CircRNAs are a class of non-coding RNAs with a covalently closed-loop structure that confers high stability in serum, making them promising biomarkers for non-invasive diagnosis and monitoring [23, 24]. Moreover, the cell- and tissue-specific expression of circRNAs across various diseases substantially augments their potential as biomarkers [25, 26]. Notably, in oncology, circRNAs hold significant promise as diagnostic and prognostic markers for a range of cancers. Accordingly, in this study, we developed a comprehensive and systematic approach for biomarker discovery and validation, aiming to identify circRNAs as liquid biopsy-based biomarkers for distinguishing GC from healthy controls and evaluating their diagnostic efficacy.

We first analyzed the circRNA profiling dataset to identify biomarkers associated with GC. Among these, the top five upregulated circRNAs in GC vs. matched paracancerous tissues were selected as initial candidates. Ultimately, hsa_circ_0001185 and hsa_circ_0005265 were identified as key circRNAs in plasma-based cohorts, enabling their translation into liquid biopsy-based diagnostic analysis. After rigorous training and validation of these circRNAs in serum, we constructed a GC diagnostic model and validated its performance in multiple, independent, serum-based clinical cohorts. We found that both circRNAs were upregulated in the serum of GC patients from different cohorts, and our model effectively distinguished GC patients in multicenter validation, exhibiting high diagnostic accuracy.

In this study, the optimal AUC values for diagnosing GC were 0.909 for hsa_circ_0001185 and 0.853 for hsa_circ_0005265. To enhance diagnostic accuracy, we conducted an integrated ROC analysis of the two circRNAs, achieving a peak AUC of 0.928 across multiple cohorts. This combined approach demonstrated superior sensitivity and specificity compared to individual biomarkers. Furthermore, the identified circRNA biomarkers demonstrated superior diagnostic performance compared to classical tumor markers. The combination of these circRNAs with conventional tumor markers enhanced the overall diagnostic accuracy for GC patients, achieving a maximum AUC of 0.963. This integrated analysis underscores the potential of these circular RNAs as robust biomarkers for GC diagnosis. In the clinically relevant GC versus gastritis comparison, the two circRNAs showed moderate discriminatory performance as individual markers, which improved when combined with conventional tumor markers. Gastritis and gastric cancer are often regarded as successive phases of a continuous disease process, in which persistent mucosal inflammation drives repeated epithelial injury-repair cycles and gradually reprograms the local microenvironment, creating conditions that facilitate malignant transformation [15]. In this context, the elevated serum levels of the two circRNAs in the gastritis group relative to healthy controls may reflect inflammation-associated mucosal stress, immune activation, and transcriptional reprogramming, whereas their further increase in gastric cancer suggests additional tumor-related influences, including sustained proliferative signaling, invasion-associated remodeling, and a chronically activated tumor microenvironment. Together, the stepwise pattern, from healthy controls to gastritis and then to gastric cancer, supports these circulating circRNAs as progression-linked liquid-biopsy signals for risk stratification and adjunctive detection of gastric cancer.

This study revealed that elevated circRNA expression was significantly correlated with lymph node metastasis, tumor invasiveness, and differentiation of GC, indicating that aberrant circRNA expression may constitute a pivotal mechanism affecting GC prognosis. Previous studies have also supported the regulatory role of circRNAs in GC. For example, CM-248aa, encoded by circular MTHFD2L RNA, inhibits GC progression by modulating the SET-PP2A interaction [27]. Additionally, exosomal circSHKBP1 drives GC progression via modulation of the miR-582-3p/HUR/VEGF axis [28]. Exosomal hsa_circ_0000437 facilitates GC metastasis via the HSPA2-ERK signaling axis [29]. These findings suggest that circular RNAs play multifaceted regulatory roles in the pathological processes of GC. Regarding the biological function of circRNAs in this study, previous literature has reported that hsa_circ_0005265 is downregulated in both the synovium and the infrapatellar fat pad (IPFP) [30]. However, research on the role of hsa_circ_0001185 and hsa_circ_0005265 as biomarkers regulating GC development has not yet been reported, suggesting that the identified circRNAs may offer new insights into GC progression. Furthermore, earlier diagnostic biomarkers faced challenges, such as low sensitivity, and the reliance on single-center cohorts, which limited their representativeness. To address these issues, we developed a predictive model for GC via logistic regression, which demonstrated high sensitivity and specificity in distinguishing potential GC patients. We improved diagnostic accuracy and screening efficiency by integrating analysis and model construction, thereby addressing the limitations of single-molecule detection. Moreover, the model was validated across multiple cohorts based on non-invasive liquid biopsy, establishing its robustness as an effective tool for distinguishing GC patients.

Our study has some limitations. First, we prioritized circRNA candidates that were significantly upregulated in GC compared with controls to facilitate assay development and potential clinical translation. However, this strategy may have overlooked downregulated circRNAs that could also be biologically informative or clinically useful [31, 32]. Second, although circRNAs are relatively stable in biofluids, circRNA dysregulation has been reported across multiple cancer types and inflammatory gastrointestinal disorders; therefore, biomarker interpretation requires evaluation against appropriate disease-control cohorts, and cancer-type specificity cannot be assumed. Because our cohorts included only GC, gastritis, and healthy individuals, we do not claim pan-gastrointestinal specificity for hsa_circ_0001185 and hsa_circ_0005265. Accordingly, we view the circRNA panel/GC-DM as a non-invasive risk-stratification tool to support clinical decision-making in patients with suspected gastric disease—complementing, rather than replacing, endoscopy—consistent with the improved discriminatory performance observed when circRNAs are integrated with conventional tumor markers. Future validation studies should incorporate additional disease controls, including other GI malignancies (e.g., colorectal, esophageal, pancreatobiliary) and systemic inflammatory conditions, to quantify cross-disease specificity and refine the intended clinical use. Third, despite encompassing clinical cohorts from multiple institutions, our study focused exclusively on Chinese patients with specific clinicopathological features, restricting the generalizability of our findings to other populations. Large-scale, prospective, multicenter international studies are warranted to confirm performance, assess clinical utility across diverse populations, and establish standardized workflows that support implementation in routine practice. Overall, while our results provide evidence supporting a circRNA-based approach for GC detection and risk stratification, additional investigations are required before these biomarkers can be considered reliable clinical assays.

In summary, we identified and validated circRNAs with the potential to serve as independent biomarkers for the detection of GC. Furthermore, we developed and validated an innovative circRNA-based predictive model for GC, demonstrating its diagnostic utility across multiple independent clinical cohorts. Our study provides new and promising evidence for the clinical application of circRNAs as non-invasive, high-throughput, and cost-effective liquid biopsy biomarkers in the detection of GC patients, with significant potential to improve cancer patient prognosis.

## Conclusions

This study identified two serum circRNAs, hsa_circ_0001185 and hsa_circ_0005265, as novel non‑invasive biomarkers for GC. We developed a multi-center diagnostic model (GC‑DM) that outperformed conventional markers in distinguishing GC from controls and gastritis patients. These findings highlighted the clinical potential of circRNA‑based liquid biopsy for GC detection.

## Supplementary Information

Supplementary Material 1.

## Data Availability

The datasets used and/or analysed during the current study are available from the corresponding author on reasonable request. We downloaded and reanalyzed the expression profile data from the Gene Expression Omnibus (GEO) database (GSE152309), accessible at https://www.ncbi.nlm.nih.gov/geo/query/acc.cgi?acc=GSE152309.

## Abbreviations

GC: Gastric cancer
circRNAs: circular RNAs
CEA: Carcinoembryonic antigen
CA199: Carbohydrate antigen 199
CA724: Carbohydrate antigen 724
RT-qPCR: Reverse Transcription Quantitative Polymerase Chain Reaction
ROC: Receiver operating characteristic
AUC: Area under the curve
CI: Confidence interval
SEN: Sensitivity
SPE: Specificity
ACCU: Overall accuracy
PPV: Positive predictive value
NPV: Negative predictive value
GC-DM: Diagnostic Model for GC
NC: Normal control
TNM: Tumor Node Metastasis
