# Supplementary material for: A Multi-Center Cohort-Based circRNA Diagnostic Model for Detection of Gastric Cancer
Source: Biol Proced Online. 2026 Jan 16;28:13. doi: 10.1186/s12575-026-00326-4 (PMC13045110; doi:10.1186/s12575-026-00326-4)
Supplement: Supplementary file 1 — Supplementary Material 1. [file 12575_2026_326_MOESM1_ESM.docx]

**Supplementary document**

**A Multi-Center Cohort-Based circRNA Diagnostic Model for Detection of Gastric Cancer**

**Running title:** A circRNA-based model for detection of Gastric Cancer.

**Xiaoyu Gu****^1^, Shuo Ma^1, 2^, Xun Gao^1^, Chenyan Yuan^1^, Wei Gao^1^, Fengfeng Zhao^1^, Yonghui Liu^1^, Chen Zhang^1^, Guoqiu Wu^1, 2, *^, Shuang Liu^1, 2, *^**

1 Center of Clinical Laboratory Medicine, Zhongda Hospital, Southeast University, Nanjing, Jiangsu, China.

2 Department of Laboratory Medicine, Medical School of Southeast University, Nanjing, Jiangsu, China.

**Corresponding authors:**

***Shuang Liu**, Center of Clinical Laboratory Medicine, Zhongda Hospital, Southeast University, Nanjing, Jiangsu, China; Department of Laboratory Medicine, Medical School of Southeast University, Nanjing, Jiangsu, China; Email: [shliu0523@163.com](mailto:shliu0523@163.com)

***Guoqiu Wu**, Center of Clinical Laboratory Medicine, Zhongda Hospital, Southeast University, Nanjing, Jiangsu, China; Department of Laboratory Medicine, Medical School of Southeast University, Nanjing, Jiangsu, China; Email: [101008404@seu.edu.cn](mailto:101008404@seu.edu.cn).

1. **Supplementary Figure legends**


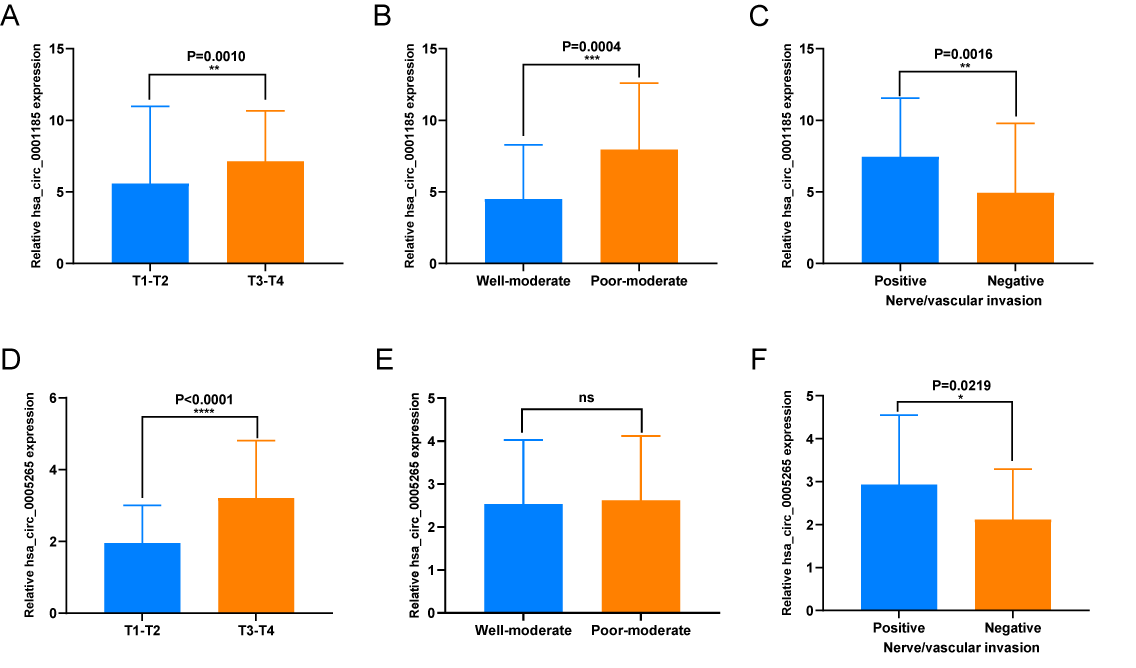


**Supplementary Figure 1.** **Clinical characteristics of  hsa_circ_0001185 and hsa_circ_0005265 in GC serum from Zhongda Hospital.** (**A and D**) The expression levels of hsa_circ_0001185 and hsa_circ_0005265 in different stages of the depth of tumor invasion. **(B and E)** The expression levels of hsa_circ_0001185 and hsa_circ_0005265 in GC patients with or without nerve/vascular invasion. **(C and F)** The expression levels of hsa_circ_0001185 and hsa_circ_0005265 in GC patients with different degrees of differentiation. *P < 0.05, **P < 0.01, ****P < 0.0001. GC, Gastric cancer.


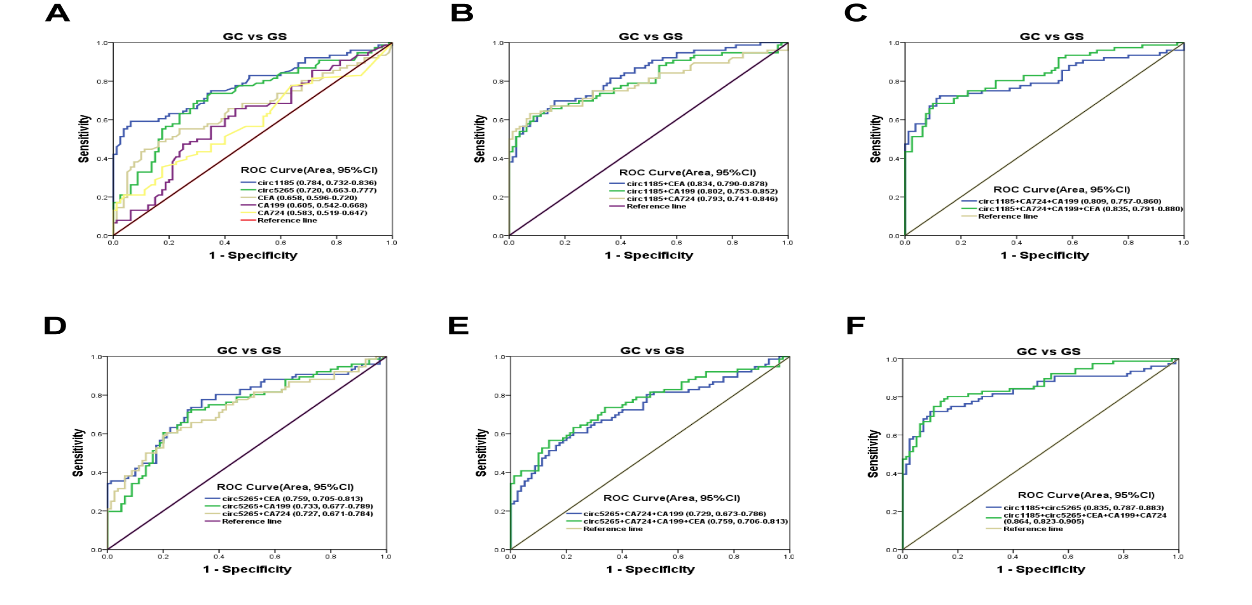


**Supplementary Figure 2. Diagnostic value of serum hsa_circ_0001185 and hsa_circ_0005265 in GC** **patients and gastritis patients. A** ROC curve analysis of hsa_circ_0001185, hsa_circ_0005265, CEA, CA199 and CA724 in independent diagnosis of GC patients and gastritis patients. **B-F** Diagnostic efficacy evaluation of combined hsa_circ_0001185 and hsa_circ_0005265 in GC patients and gastritis patients. GC, Gastric cancer; GS, Gastritis; ROC, Receiver Operating Characteristic curve; AUC, Area Under the Curve; CI, Confidence Interval.


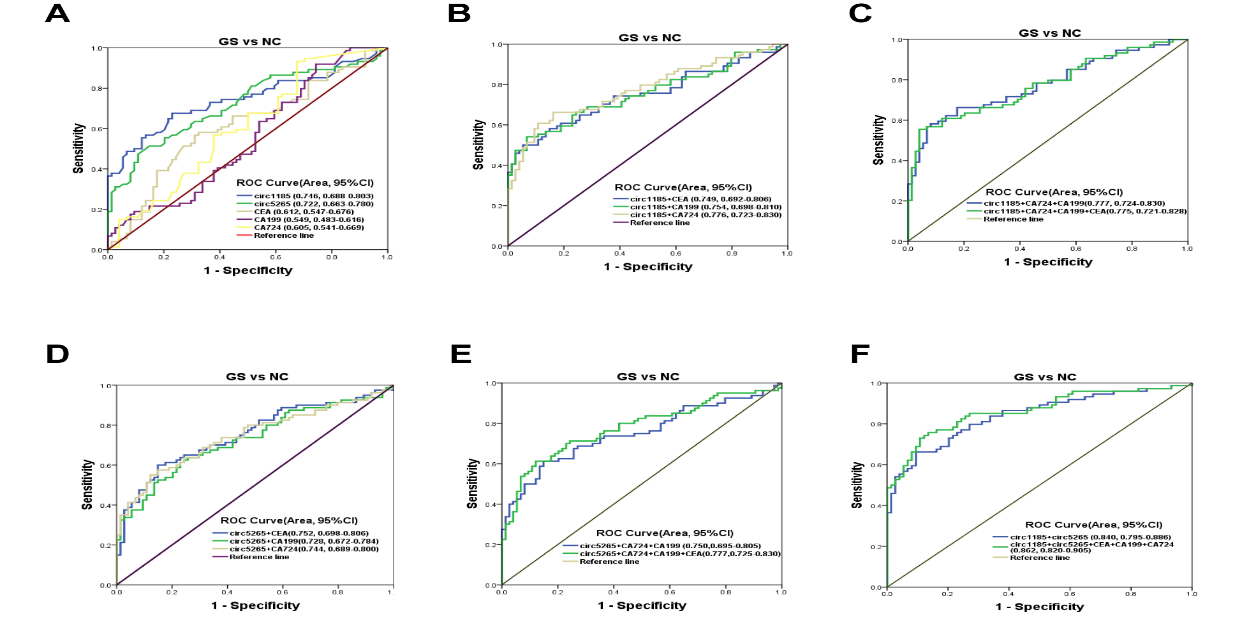


**Supplementary Figure 3. Diagnostic value of serum hsa_circ_0001185 and hsa_circ_0005265 in gastritis patients and healthy donors.** **A** ROC curve analysis of hsa_circ_0001185, hsa_circ_0005265, CEA, CA199 and CA724 in independent diagnosis of gastritis patients and healthy donors. **B-F** Diagnostic efficacy evaluation of combined hsa_circ_0001185 and hsa_circ_0005265 in gastritis patients and healthy donors. GS, Gastritis; ROC, Receiver Operating Characteristic curve; AUC, Area Under the Curve; CI, Confidence Interval.


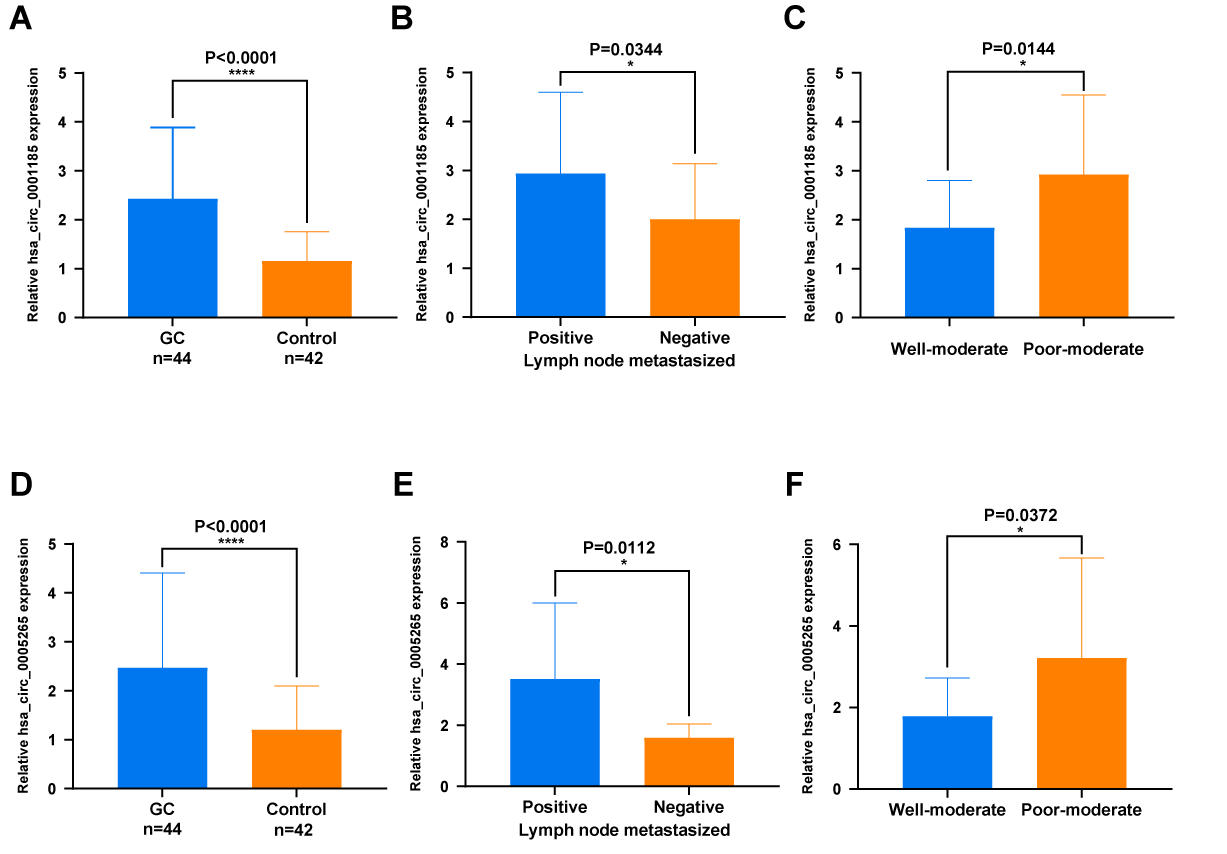


**Supplementary Figure 4. Clinical characteristics of hsa_circ_0001185 and hsa_circ-0005265 in serum of GC patients in Beijing China Japan Friendship Hospital.** (**A and D**) The expression levels of hsa_circ_0001185 and hsa_circ_0005265 in the serum of GC patients (n=44) and healthy donors (n=42). **(B and E)** The expression levels of hsa_circ_0001185 and hsa_circ_0005265 in GC patients with or without lymph node metastasis. **(C and F)** The expression levels of hsa_circ_0001185 and hsa_circ_0005265 in GC patients with different degrees of differentiation. *P < 0.05, **P < 0.01, ****P < 0.0001. GC, Gastric cancer.


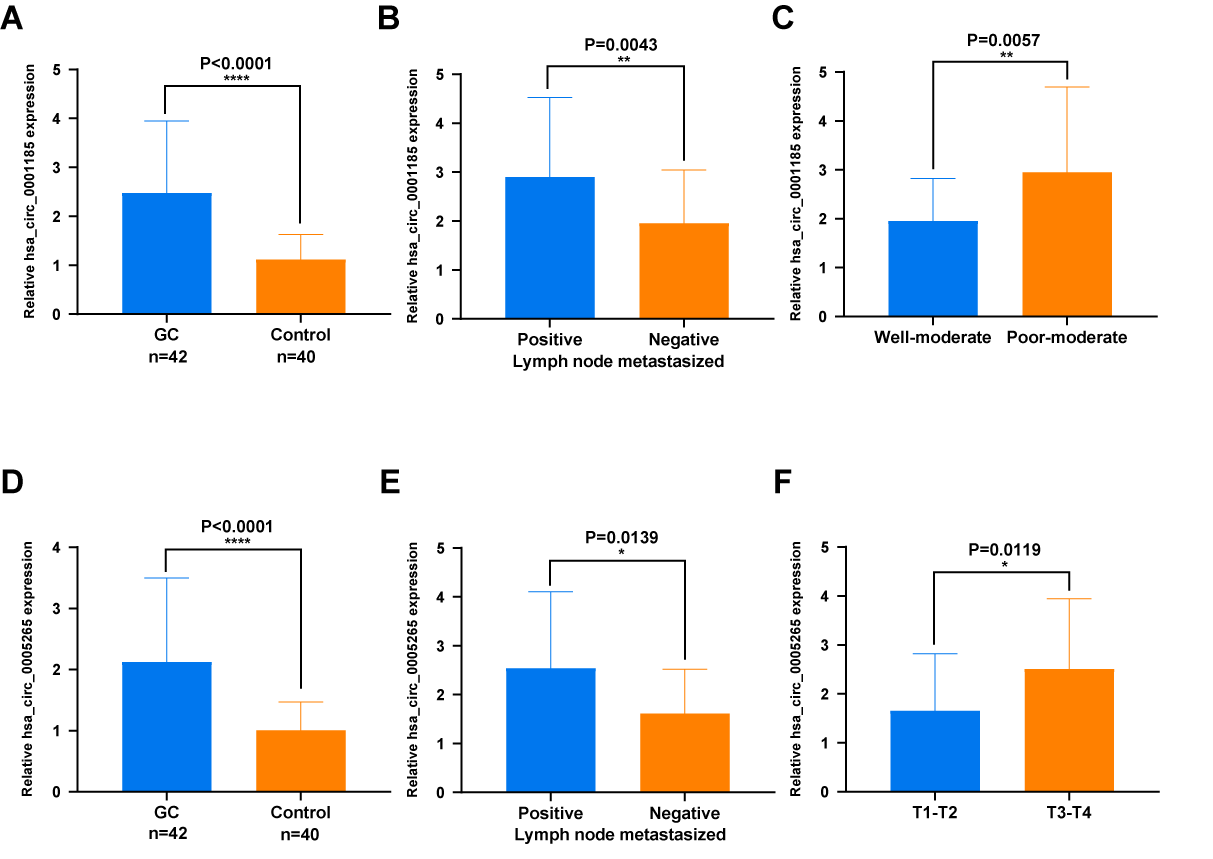


**Supplementary Figure 5. Clinical characteristics of hsa_circ_0001185 and hsa_circ-0005265 in serum of GC patients in Affiliated Hospital of Nantong University.** (**A and D**) The expression levels of hsa_circ_0001185 and hsa_circ_0005265 in the serum of GC patients (n=42) and healthy donors (n=40). **(B and E)** The expression levels of hsa_circ_0001185 and hsa_circ_0005265 in GC patients with or without lymph node metastasis. **(C and F)** The expression levels of hsa_circ_0001185 and hsa_circ_0005265 in GC patients with different degrees of differentiation. *P < 0.05, **P < 0.01, ****P < 0.0001. GC, Gastric cancer.


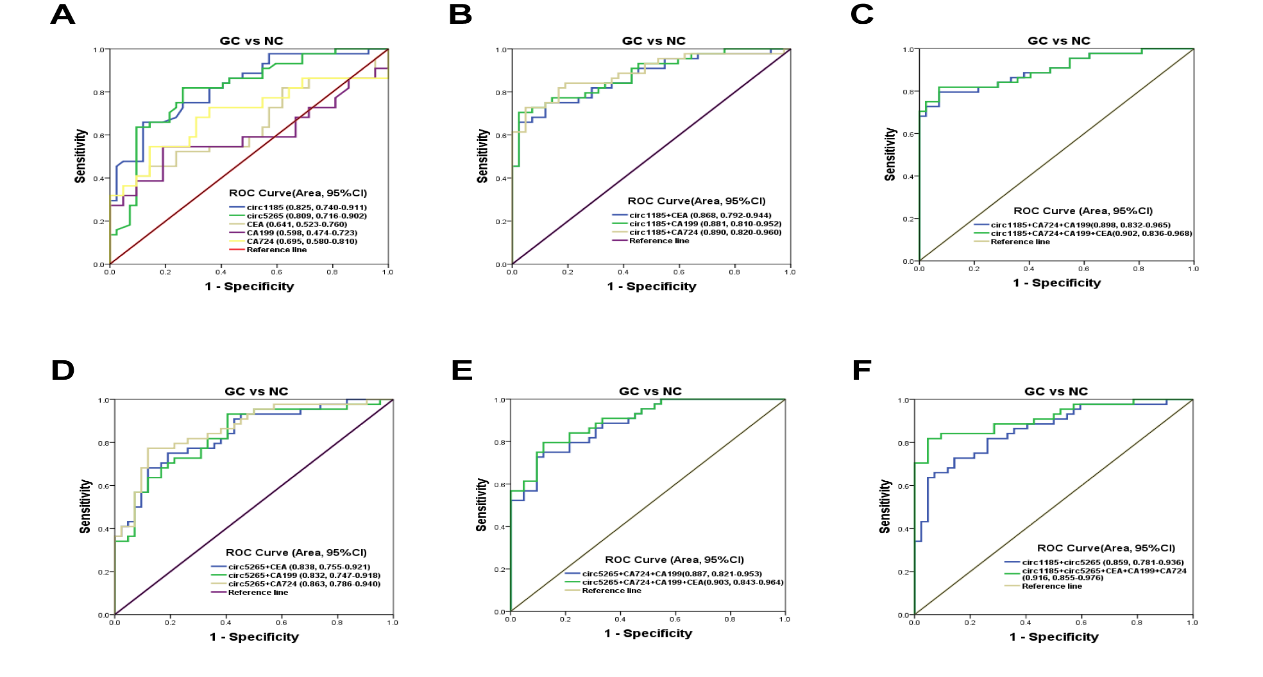


**Supplementary Figure 6. Diagnostic value of serum hsa_circ_0001185 and hsa_circ_0005265 in GC** **from Beijing-China Japan Friendship Hospital. A** ROC curve analysis of hsa_circ_0001185, hsa_circ_0005265, CEA, CA199 and CA724 in independent diagnosis of GC patients and healthy donors. **B-F** Diagnostic efficacy evaluation of combined hsa_circ_0001185 and hsa_circ_0005265 in GC patients and healthy donors. GC, Gastric cancer; ROC, Receiver Operating Characteristic curve; AUC, Area Under the Curve; CI, Confidence Interval.


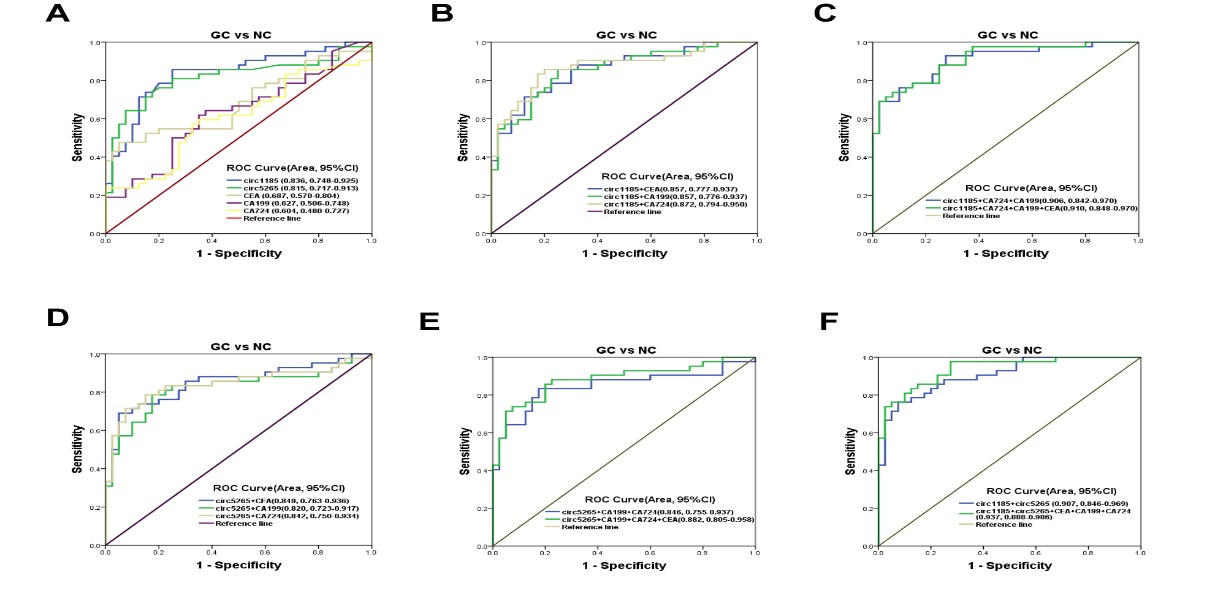


**Supplementary Figure 7. Diagnostic value of serum hsa_circ_0001185 and hsa_circ_0005265 in GC** **from Affiliated Hospital of Nantong University. A** ROC curve analysis of hsa_circ_0001185, hsa_circ_0005265, CEA, CA199 and CA724 in independent diagnosis of GC patients and healthy donors. **B-F** Diagnostic efficacy evaluation of combined hsa_circ_0001185 and hsa_circ_0005265 in GC patients and healthy donors. GC, Gastric cancer; ROC, Receiver Operating Characteristic curve; AUC, Area Under the Curve; CI, Confidence Interval.

| **Supplementary Table 1. The diagnostic performance of circ1185, circ5265, CEA, CA199, and CA724 in differentiating GC patients from Gastritis donors.** | | | | | |
| --- | --- | --- | --- | --- | --- |
|  | **SEN** | **SPE** | **ACCU** | **PPV** | **NPV** |
| circ1185 | 0.59 (90/152) | 0.93 (148/160) | 0.76 (238/312) | 0.88 (90/102) | 0.70 (148/210) |
| circ5265 | 0.70 (106/152) | 0.70 (112/160) | 0.70 (218/312) | 0.69 (106/154) | 0.71 (112/158) |
| CEA | 0.45 (68/152) | 0.88 (141/160) | 0.67 (209/312) | 0.78 (68/87) | 0.63 (141/225) |
| CA199 | 0.47 (72/152) | 0.75 (120/160) | 0.62 (192/312) | 0.64 (72/112) | 0.60 (120/200) |
| CA724 | 0.38 (58/152) | 0.83 (132/160) | 0.61 (190/312) | 0.67 (58/86) | 0.58 (132/226) |
| circ1185+CEA | 0.70 (106/152) | 0.84 (134/160) | 0.77 (240/312) | 0.80 (106/132) | 0.74 (134/180) |
| circ1185+CA199 | 0.64 (98/152) | 0.88 (140/160) | 0.76 (238/312) | 0.88 (98/112) | 0.72 (140/194) |
| circ1185+CA724 | 0.63 (96/152) | 0.91 (146/160) | 0.78 (242/312) | 0.87 (96/110) | 0.72 (146/202) |
| circ1185+CA724+CA199 | 0.72 (110/152) | 0.88 (140/160) | 0.80 (250/312) | 0.85 (110/130) | 0.77 (140/182) |
| circ1185+CA724+CA199+CEA | 0.68 (104/152) | 0.89 (143/160) | 0.79 (247/312) | 0.86 (104/121) | 0.75 (143/191) |
| circ5265+CEA | 0.78 (118/152) | 0.66 (106/160) | 0.72 (224/312) | 0.69 (118/172) | 0.76 (106/140) |
| circ5265+CA199 | 0.72 (110/152) | 0.70 (112/160) | 0.71 (222/312) | 0.70 (110/158) | 0.73 (112/154) |
| circ5265+CA724 | 0.61 (92/152) | 0.79 (126/160) | 0.70 (218/312) | 0.73 (92/126) | 0.68(126/186) |
| circ5265+CA724+CA199 | 0.61 (92/152) | 0.78 (124/160) | 0.69 (216/312) | 0.72 (92/128) | 0.67 (124/184) |
| circ5265+CA724+CA199+CEA | 0.57 (86/152) | 0.86 (138/160) | 0.72 (224/312) | 0.80 (86/108) | 0.68 (138/204) |
| circ1185+circ5265 | 0.71 (108/152) | 0.90 (144/160) | 0.81 (252/312) | 0.87 (108/124) | 0.77 (144/188) |
| circ1185+circ5265+CEA+CA199+CA724 | 0.80 (122/152) | 0.84 (134/160) | 0.82 (256/312) | 0.82 (122/148) | 0.82 (134/164) |

1. **Supplementary Tables**

GC, gastric cancer; circ, circular; CA, carbohydrate antigen; CEA, carcinoembryonic antigen; SEN, sensitivity; SPE, specificity; ACCU, overall accuracy; NPV, negative predictive value; PPV, positive predictive value.

| **Supplementary Table 2. The diagnostic performance of circ1185, CEA, CA199, and CA724 in differentiating**  **Gastritis patients from healthy donors** | | | | | | | | | | | | | | | | | | | | | | | | | | | | | | | | | | | | | | | | | | | | | | | | | | | | | | | | | | | | | | |
| --- | --- | --- | --- | --- | --- | --- | --- | --- | --- | --- | --- | --- | --- | --- | --- | --- | --- | --- | --- | --- | --- | --- | --- | --- | --- | --- | --- | --- | --- | --- | --- | --- | --- | --- | --- | --- | --- | --- | --- | --- | --- | --- | --- | --- | --- | --- | --- | --- | --- | --- | --- | --- | --- | --- | --- | --- | --- | --- | --- | --- | --- | --- |
|  | **SEN** | **SPE** | **ACCU** | **PPV** | **NPV** |  |  |  |  |  |  |  |  |  |  |  |  |  |  |  |  |  |  |  |  |  |  |  |  |  |  |  |  |  |  |  |  |  |  |  |  |  |  |  |  |  |  |  |  |  |  |  |  |  |  |  |  |  |  |  |  |  |
| circ1185 | 0.69 (110/160) | 0.77 (114/148) | 0.73 (224/308) | 0.76 (110/144) | 0.70 (114/164) |  |  |  |  |  |  |  |  |  |  |  |  |  |  |  |  |  |  |  |  |  |  |  |  |  |  |  |  |  |  |  |  |  |  |  |  |  |  |  |  |  |  |  |  |  |  |  |  |  |  |  |  |  |  |  |  |  |
| circ5265 | 0.48 (76/160) | 0.89 (132/148) | 0.68 (208/308) | 0.83 (76/92) | 0.61 (132/216) |  |  |  |  |  |  |  |  |  |  |  |  |  |  |  |  |  |  |  |  |  |  |  |  |  |  |  |  |  |  |  |  |  |  |  |  |  |  |  |  |  |  |  |  |  |  |  |  |  |  |  |  |  |  |  |  |  |
| CEA | 0.58 (92/160) | 0.69 (102/148) | 0.63 (194/308) | 0.67 (92/138) | 0.60 (102/170) |  |  |  |  |  |  |  |  |  |  |  |  |  |  |  |  |  |  |  |  |  |  |  |  |  |  |  |  |  |  |  |  |  |  |  |  |  |  |  |  |  |  |  |  |  |  |  |  |  |  |  |  |  |  |  |  |  |
| CA199 | 0.91 (146/160) | 0.26 (38/148) | 0.60 (184/308) | 0.57 (146/256) | 0.73 (38/52) |  |  |  |  |  |  |  |  |  |  |  |  |  |  |  |  |  |  |  |  |  |  |  |  |  |  |  |  |  |  |  |  |  |  |  |  |  |  |  |  |  |  |  |  |  |  |  |  |  |  |  |  |  |  |  |  |  |
| CA724 | 0.88 (140/160) | 0.32 (48/148) | 0.61 (188/308) | 0.58 (140/240) | 0.71 (48/68) |  |  |  |  |  |  |  |  |  |  |  |  |  |  |  |  |  |  |  |  |  |  |  |  |  |  |  |  |  |  |  |  |  |  |  |  |  |  |  |  |  |  |  |  |  |  |  |  |  |  |  |  |  |  |  |  |  |
| circ1185+CEA | 0.49 (78/160) | 0.93 (138/148) | 0.70 (216/308) | 0.89 (78/88) | 0.63 (138/220) |  |  |  |  |  |  |  |  |  |  |  |  |  |  |  |  |  |  |  |  |  |  |  |  |  |  |  |  |  |  |  |  |  |  |  |  |  |  |  |  |  |  |  |  |  |  |  |  |  |  |  |  |  |  |  |  |  |
| circ1185+CA199 | 0.53 (84/160) | 0.93 (138/148) | 0.72 (222/308) | 0.89 (84/94) | 0.64 (138/214) |  |  |  |  |  |  |  |  |  |  |  |  |  |  |  |  |  |  |  |  |  |  |  |  |  |  |  |  |  |  |  |  |  |  |  |  |  |  |  |  |  |  |  |  |  |  |  |  |  |  |  |  |  |  |  |  |  |
| circ1185+CA724 | 0.56 (90/160) | 0.89 (132/148) | 0.72 (222/308) | 0.85 (90/106) | 0.65 (132/202) |  |  |  |  |  |  |  |  |  |  |  |  |  |  |  |  |  |  |  |  |  |  |  |  |  |  |  |  |  |  |  |  |  |  |  |  |  |  |  |  |  |  |  |  |  |  |  |  |  |  |  |  |  |  |  |  |  |
| circ1185+CA724+CA199 | 0.53 (84/160) | 0.93 (138/148) | 0.72 (222/308) | 0.89 (84/94) | 0.64 (138/214) |  |  |  |  |  |  |  |  |  |  |  |  |  |  |  |  |  |  |  |  |  |  |  |  |  |  |  |  |  |  |  |  |  |  |  |  |  |  |  |  |  |  |  |  |  |  |  |  |  |  |  |  |  |  |  |  |  |
| circ1185+CA724+CA199+CEA | 0.51 (82/160) | 0.96 (142/148) | 0.73 (224/308) | 0.93 (82/88) | 0.65 (142/220) |  |  |  |  |  |  |  |  |  |  |  |  |  |  |  |  |  |  |  |  |  |  |  |  |  |  |  |  |  |  |  |  |  |  |  |  |  |  |  |  |  |  |  |  |  |  |  |  |  |  |  |  |  |  |  |  |  |
| circ5265+CEA | 0.60 (96/160) | 0.85 (126/148) | 0.72 (222/308) | 0.81 (96/118) | 0.66 (126/190) |  |  |  |  |  |  |  |  |  |  |  |  |  |  |  |  |  |  |  |  |  |  |  |  |  |  |  |  |  |  |  |  |  |  |  |  |  |  |  |  |  |  |  |  |  |  |  |  |  |  |  |  |  |  |  |  |  |
| circ5265+CA199 | 0.61 (98/160) | 0.76 (112/148) | 0.68 (210/308) | 0.73 (98/134) | 0.64 (112/174) |  |  |  |  |  |  |  |  |  |  |  |  |  |  |  |  |  |  |  |  |  |  |  |  |  |  |  |  |  |  |  |  |  |  |  |  |  |  |  |  |  |  |  |  |  |  |  |  |  |  |  |  |  |  |  |  |  |
| circ5265+CA724 | 0.55 (88/160) | 0.86 (128/148) | 0.70 (216/308) | 0.81 (88/108) | 0.64 (128/200) |  |  |  |  |  |  |  |  |  |  |  |  |  |  |  |  |  |  |  |  |  |  |  |  |  |  |  |  |  |  |  |  |  |  |  |  |  |  |  |  |  |  |  |  |  |  |  |  |  |  |  |  |  |  |  |  |  |
| circ5265+CA724+CA199 | 0.61 (98/160) | 0.84 (124/148) | 0.72 (222/308) | 0.80 (98/122) | 0.67 (124/186) |  |  |  |  |  |  |  |  |  |  |  |  |  |  |  |  |  |  |  |  |  |  |  |  |  |  |  |  |  |  |  |  |  |  |  |  |  |  |  |  |  |  |  |  |  |  |  |  |  |  |  |  |  |  |  |  |  |
| circ5265+CA724+CA199+CEA | 0.61 (98/160) | 0.88 (130/148) | 0.74 (228/308) | 0.84 (98/116) | 0.68 (130/192) |  |  |  |  |  |  |  |  |  |  |  |  |  |  |  |  |  |  |  |  |  |  |  |  |  |  |  |  |  |  |  |  |  |  |  |  |  |  |  |  |  |  |  |  |  |  |  |  |  |  |  |  |  |  |  |  |  |
| circ1185+circ5265 | 0.66 (106/160) | 0.91 (134/148) | 0.78 (240/308) | 0.88 (106/120) | 0.71 (134/188) |  |  |  |  |  |  |  |  |  |  |  |  |  |  |  |  |  |  |  |  |  |  |  |  |  |  |  |  |  |  |  |  |  |  |  |  |  |  |  |  |  |  |  |  |  |  |  |  |  |  |  |  |  |  |  |  |  |
| circ1185+circ5265+CEA+CA199+CA724 | 0.68 (108/160) | 0.89 (132/148) | 0.78 (240/308) | 0.87 (108/124) | 0.72 (132/184) |  |  |  |  |  |  |  |  |  |  |  |  |  |  |  |  |  |  |  |  |  |  |  |  |  |  |  |  |  |  |  |  |  |  |  |  |  |  |  |  |  |  |  |  |  |  |  |  |  |  |  |  |  |  |  |  |  |

GC, gastric cancer; circ, circular; CA, carbohydrate antigen; CEA, carcinoembryonic antigen; SEN, sensitivity; SPE, specificity; ACCU, overall accuracy; NPV, negative predictive value; PPV, positive predictive value.

**Supplementary Table 3. Clinicopathological analysis of hsa_circ_0001185 in GC from China-Japan Friendship Hospital.**

| **Parameter** |  | **No. of patients** | **hsa_circ_000**  **1185（high）** | **hsa_circ_000**  **1185（low）** | **P-value** |
| --- | --- | --- | --- | --- | --- |
| Sex | Male | 30 | 14 | 16 | 0.5174 |
|  | Female | 14 | 8 | 6 |  |
| Age（years） | ≤60 | 18 | 8 | 10 | 0.6857 |
|  | ＞60 | 26 | 14 | 12 |  |
| Grade | Well-moderate | 19 | 6 | 13 | 0.0331^*^ |
|  | Poor-undifferentiation | 25 | 16 | 9 |  |
| Pathologic type | Adenocarcinoma | 38 | 18 | 20 | 0.1066 |
|  | Adenosquamous carcinoma | 2 | 0 | 2 |  |
|  | Signet-ring cell carcinoma | 2 | 2 | 0 |  |
|  | Mucinous adenocarcinoma | 2 | 2 | 0 |  |
| Lymph node status | Positive | 20 | 12 | 8 | 0.2259 |
|  | Negative | 24 | 10 | 14 |  |
| TNM stage | Ⅰ-Ⅱ | 25 | 10 | 15 | 0.1281 |
|  | Ⅲ-Ⅳ | 19 | 12 | 7 |  |
| Ki67 | Positive | 24 | 16 | 8 | 0.0154^*^ |
|  | Negative | 20 | 6 | 14 |  |
| CK | Positive | 22 | 15 | 7 | 0.0159^*^ |
|  | Negative | 22 | 7 | 15 |  |

circ, circular; GC, gastric cancer; TNM, tumor node metastasis

*P < 0.05, **P < 0.01, ***P < 0.001, ****P < 0.0001.

**Supplementary Table 4. Clinicopathological analysis of hsa_circ_0005265 in GC from China-Japan Friendship Hospital.**

| **Parameter** |  | **No. of patients** | **hsa_circ_000**  **5265（high）** | **hsa_circ_000**  **5265（low）** | **P-value** |
| --- | --- | --- | --- | --- | --- |
| Sex | Male | 30 | 17 | 13 | 0.1954 |
|  | Female | 14 | 5 | 9 |  |
| Age（years） | ≤60 | 18 | 9 | 9 | >0.9999 |
|  | ＞60 | 26 | 13 | 13 |  |
| Grade | Well-moderate | 19 | 8 | 11 | 0.3612 |
|  | Poor-undifferentiation | 25 | 14 | 11 |  |
| Pathologic type | Adenocarcinoma | 38 | 17 | 21 | 0.2194 |
|  | Adenosquamous carcinoma | 2 | 2 | 0 |  |
|  | Signet-ring cell carcinoma | 2 | 1 | 1 |  |
|  | Mucinous adenocarcinoma | 2 | 2 | 0 |  |
| Lymph node status | Positive | 20 | 14 | 6 | 0.0154^*^ |
|  | Negative | 24 | 8 | 16 |  |
| TNM stage | Ⅰ-Ⅱ | 25 | 12 | 13 | 0.7609 |
|  | Ⅲ-Ⅳ | 19 | 10 | 9 |  |
| Ki67 | Positive | 24 | 16 | 8 | 0.0154^*^ |
|  | Negative | 20 | 6 | 14 |  |
| CK | Positive | 22 | 14 | 8 | 0.0704 |
|  | Negative | 22 | 8 | 14 |  |

circ, circular; GC, gastric cancer; TNM, tumor node metastasis

*P < 0.05, **P < 0.01, ***P < 0.001, ****P < 0.0001.

| **Supplementary Table 5. Clinicopathological analysis of hsa_circ_0001185 in GC from Affiliated**  **Hospital of Nantong University.** | | | | | | | | | | | | | | | | | | | | | | | | | | | | | | | | | | | | | | | | | | | | | | | | | | | | | | | | | | | | | | |
| --- | --- | --- | --- | --- | --- | --- | --- | --- | --- | --- | --- | --- | --- | --- | --- | --- | --- | --- | --- | --- | --- | --- | --- | --- | --- | --- | --- | --- | --- | --- | --- | --- | --- | --- | --- | --- | --- | --- | --- | --- | --- | --- | --- | --- | --- | --- | --- | --- | --- | --- | --- | --- | --- | --- | --- | --- | --- | --- | --- | --- | --- | --- |
| **Parameter** |  | **No. of patients** | **hsa_circ_**  **0001185（high）** | **hsa_circ_**  **0001185（low）** | **P-value** |  |  |  |  |  |  |  |  |  |  |  |  |  |  |  |  |  |  |  |  |  |  |  |  |  |  |  |  |  |  |  |  |  |  |  |  |  |  |  |  |  |  |  |  |  |  |  |  |  |  |  |  |  |  |  |  |  |
| Sex | Male | 31 | 15 | 16 | 0.7256 |  |  |  |  |  |  |  |  |  |  |  |  |  |  |  |  |  |  |  |  |  |  |  |  |  |  |  |  |  |  |  |  |  |  |  |  |  |  |  |  |  |  |  |  |  |  |  |  |  |  |  |  |  |  |  |  |  |
|  | Female | 11 | 6 | 5 |  |  |  |  |  |  |  |  |  |  |  |  |  |  |  |  |  |  |  |  |  |  |  |  |  |  |  |  |  |  |  |  |  |  |  |  |  |  |  |  |  |  |  |  |  |  |  |  |  |  |  |  |  |  |  |  |  |  |
| Age（years） | ≤60 | 15 | 8 | 7 | 0.7474 |  |  |  |  |  |  |  |  |  |  |  |  |  |  |  |  |  |  |  |  |  |  |  |  |  |  |  |  |  |  |  |  |  |  |  |  |  |  |  |  |  |  |  |  |  |  |  |  |  |  |  |  |  |  |  |  |  |
|  | ＞60 | 27 | 13 | 14 |  |  |  |  |  |  |  |  |  |  |  |  |  |  |  |  |  |  |  |  |  |  |  |  |  |  |  |  |  |  |  |  |  |  |  |  |  |  |  |  |  |  |  |  |  |  |  |  |  |  |  |  |  |  |  |  |  |  |
| Grade | Well-moderate | 22 | 9 | 13 | 0.2165 |  |  |  |  |  |  |  |  |  |  |  |  |  |  |  |  |  |  |  |  |  |  |  |  |  |  |  |  |  |  |  |  |  |  |  |  |  |  |  |  |  |  |  |  |  |  |  |  |  |  |  |  |  |  |  |  |  |
|  | Poor-undifferentiation | 20 | 12 | 8 |  |  |  |  |  |  |  |  |  |  |  |  |  |  |  |  |  |  |  |  |  |  |  |  |  |  |  |  |  |  |  |  |  |  |  |  |  |  |  |  |  |  |  |  |  |  |  |  |  |  |  |  |  |  |  |  |  |  |
| Pathologic type | Adenocarcinoma | 40 | 19 | 21 | 0.35 |  |  |  |  |  |  |  |  |  |  |  |  |  |  |  |  |  |  |  |  |  |  |  |  |  |  |  |  |  |  |  |  |  |  |  |  |  |  |  |  |  |  |  |  |  |  |  |  |  |  |  |  |  |  |  |  |  |
|  | Signet-ring cell carcinoma | 1 | 1 | 0 |  |  |  |  |  |  |  |  |  |  |  |  |  |  |  |  |  |  |  |  |  |  |  |  |  |  |  |  |  |  |  |  |  |  |  |  |  |  |  |  |  |  |  |  |  |  |  |  |  |  |  |  |  |  |  |  |  |  |
|  | Mucinous adenocarcinoma | 1 | 1 | 0 |  |  |  |  |  |  |  |  |  |  |  |  |  |  |  |  |  |  |  |  |  |  |  |  |  |  |  |  |  |  |  |  |  |  |  |  |  |  |  |  |  |  |  |  |  |  |  |  |  |  |  |  |  |  |  |  |  |  |
| Lymph node status | Positive | 25 | 13 | 12 | 0.7532 |  |  |  |  |  |  |  |  |  |  |  |  |  |  |  |  |  |  |  |  |  |  |  |  |  |  |  |  |  |  |  |  |  |  |  |  |  |  |  |  |  |  |  |  |  |  |  |  |  |  |  |  |  |  |  |  |  |
|  | Negative | 17 | 8 | 9 |  |  |  |  |  |  |  |  |  |  |  |  |  |  |  |  |  |  |  |  |  |  |  |  |  |  |  |  |  |  |  |  |  |  |  |  |  |  |  |  |  |  |  |  |  |  |  |  |  |  |  |  |  |  |  |  |  |  |
| TNM stage | Ⅰ-Ⅱ | 19 | 9 | 10 | 0.7565 |  |  |  |  |  |  |  |  |  |  |  |  |  |  |  |  |  |  |  |  |  |  |  |  |  |  |  |  |  |  |  |  |  |  |  |  |  |  |  |  |  |  |  |  |  |  |  |  |  |  |  |  |  |  |  |  |  |
|  | Ⅲ-Ⅳ | 23 | 12 | 11 |  |  |  |  |  |  |  |  |  |  |  |  |  |  |  |  |  |  |  |  |  |  |  |  |  |  |  |  |  |  |  |  |  |  |  |  |  |  |  |  |  |  |  |  |  |  |  |  |  |  |  |  |  |  |  |  |  |  |
| Nerve/vascular invasion | Positive | 23 | 17 | 6 | 0.0006^***^ |  |  |  |  |  |  |  |  |  |  |  |  |  |  |  |  |  |  |  |  |  |  |  |  |  |  |  |  |  |  |  |  |  |  |  |  |  |  |  |  |  |  |  |  |  |  |  |  |  |  |  |  |  |  |  |  |  |
|  | Negative | 19 | 4 | 15 |  |  |  |  |  |  |  |  |  |  |  |  |  |  |  |  |  |  |  |  |  |  |  |  |  |  |  |  |  |  |  |  |  |  |  |  |  |  |  |  |  |  |  |  |  |  |  |  |  |  |  |  |  |  |  |  |  |  |
| Depth of infiltration | Negative | 20 | 6 | 14 | 0.0134^*^ |  |  |  |  |  |  |  |  |  |  |  |  |  |  |  |  |  |  |  |  |  |  |  |  |  |  |  |  |  |  |  |  |  |  |  |  |  |  |  |  |  |  |  |  |  |  |  |  |  |  |  |  |  |  |  |  |  |
|  | Submucosa and above | 22 | 15 | 7 |  |  |  |  |  |  |  |  |  |  |  |  |  |  |  |  |  |  |  |  |  |  |  |  |  |  |  |  |  |  |  |  |  |  |  |  |  |  |  |  |  |  |  |  |  |  |  |  |  |  |  |  |  |  |  |  |  |  |

circ, circular; GC, gastric cancer; TNM, tumor node metastasis

*P < 0.05, **P < 0.01, ***P < 0.001, ****P < 0.0001.

**Supplementary Table 6. Clinicopathological analysis of hsa_circ_0005265 in GC from** **Affiliated Hospital of Nantong University.**

| **Parameter** |  | **No.of patients** | **hsa_circ_000**  **5265（high）** | **hsa_circ_000**  **5265（low）** | **P-value** |
| --- | --- | --- | --- | --- | --- |
| Sex | Male | 31 | 14 | 17 | 0.2924 |
|  | Female | 11 | 7 | 4 |  |
| Age（years） | ≤60 | 15 | 7 | 8 | 0.7474 |
|  | ＞60 | 27 | 14 | 13 |  |
| Grade | Well-moderate | 22 | 8 | 14 | 0.0638 |
|  | Poor-undifferentiation | 20 | 13 | 7 |  |
| Pathologic type | Adenocarcinoma | 40 | 19 | 21 | 0.3662 |
|  | Signet-ring cell carcinoma | 1 | 1 | 0 |  |
|  | Mucinous adenocarcinoma | 1 | 0 | 1 |  |
| Lymph node status | Positive | 25 | 14 | 11 | 0.3456 |
|  | Negative | 17 | 7 | 10 |  |
| TNM stage | Ⅰ-Ⅱ | 19 | 6 | 13 | 0.0300^*^ |
|  | Ⅲ-Ⅳ | 23 | 15 | 8 |  |
| Nerve/vascular invasion | Positive | 23 | 10 | 13 | 0.3523 |
|  | Negative | 19 | 11 | 8 |  |
| Depth of infiltration | Negative | 20 | 11 | 9 | 0.5366 |
|  | submucosa and above | 22 | 10 | 12 |  |

circ, circular; GC, gastric cancer; TNM, tumor node metastasis

*P < 0.05, **P < 0.01, ***P < 0.001, ****P < 0.0001.

| **Supplementary Table 7. The diagnostic performance of circ1185, CEA, CA199, and CA724 in differentiating**  **GC patients from healthy donors in** **China-Japan Friendship Hospital.** | | | | | | | | | | | | | | | | | | | | | | | | | | | | | | | | | | | | | | | | | | | | | | | | | | | | | | | | | | | | | | |
| --- | --- | --- | --- | --- | --- | --- | --- | --- | --- | --- | --- | --- | --- | --- | --- | --- | --- | --- | --- | --- | --- | --- | --- | --- | --- | --- | --- | --- | --- | --- | --- | --- | --- | --- | --- | --- | --- | --- | --- | --- | --- | --- | --- | --- | --- | --- | --- | --- | --- | --- | --- | --- | --- | --- | --- | --- | --- | --- | --- | --- | --- | --- |
|  | **SEN** | **SPE** | **ACCU** | **PPV** | **NPV** |  |  |  |  |  |  |  |  |  |  |  |  |  |  |  |  |  |  |  |  |  |  |  |  |  |  |  |  |  |  |  |  |  |  |  |  |  |  |  |  |  |  |  |  |  |  |  |  |  |  |  |  |  |  |  |  |  |
| circ1185 | 0.66 (29/44) | 0.88 (37/42) | 0.77 (66/86) | 0.85 (29/34) | 0.71 (37/52) |  |  |  |  |  |  |  |  |  |  |  |  |  |  |  |  |  |  |  |  |  |  |  |  |  |  |  |  |  |  |  |  |  |  |  |  |  |  |  |  |  |  |  |  |  |  |  |  |  |  |  |  |  |  |  |  |  |
| circ5265 | 0.82 (36/44) | 0.74 (31/42) | 0.78 (67/86) | 0.77 (36/47) | 0.79 (31/39) |  |  |  |  |  |  |  |  |  |  |  |  |  |  |  |  |  |  |  |  |  |  |  |  |  |  |  |  |  |  |  |  |  |  |  |  |  |  |  |  |  |  |  |  |  |  |  |  |  |  |  |  |  |  |  |  |  |
| CEA | 0.41 (18/44) | 0.90 (38/42) | 0.65 (56/86) | 0.82 (18/22) | 0.59 (38/64) |  |  |  |  |  |  |  |  |  |  |  |  |  |  |  |  |  |  |  |  |  |  |  |  |  |  |  |  |  |  |  |  |  |  |  |  |  |  |  |  |  |  |  |  |  |  |  |  |  |  |  |  |  |  |  |  |  |
| CA199 | 0.55 (24/44) | 0.81 (34/42) | 0.71 (61/86) | 0.78 (25/31) | 0.65 (36/55) |  |  |  |  |  |  |  |  |  |  |  |  |  |  |  |  |  |  |  |  |  |  |  |  |  |  |  |  |  |  |  |  |  |  |  |  |  |  |  |  |  |  |  |  |  |  |  |  |  |  |  |  |  |  |  |  |  |
| CA724 | 0.57 (25/44) | 0.86 (36/42) | 0.67 (58/86) | 0.75 (24/32) | 0.63 (34/54) |  |  |  |  |  |  |  |  |  |  |  |  |  |  |  |  |  |  |  |  |  |  |  |  |  |  |  |  |  |  |  |  |  |  |  |  |  |  |  |  |  |  |  |  |  |  |  |  |  |  |  |  |  |  |  |  |  |
| circ1185+CEA | 0.66 (29/44) | 0.95 (40/42) | 0.80 (69/86) | 0.94 (29/31) | 0.73 (40/55) |  |  |  |  |  |  |  |  |  |  |  |  |  |  |  |  |  |  |  |  |  |  |  |  |  |  |  |  |  |  |  |  |  |  |  |  |  |  |  |  |  |  |  |  |  |  |  |  |  |  |  |  |  |  |  |  |  |
| circ1185+CA199 | 0.70 (31/44) | 0.95 (40/42) | 0.83 (71/86) | 0.94 (31/33) | 0.75 (40/53) |  |  |  |  |  |  |  |  |  |  |  |  |  |  |  |  |  |  |  |  |  |  |  |  |  |  |  |  |  |  |  |  |  |  |  |  |  |  |  |  |  |  |  |  |  |  |  |  |  |  |  |  |  |  |  |  |  |
| circ1185+CA724 | 0.73 (32/44) | 0.95 (40/42) | 0.84 (72/86) | 0.94 (32/34) | 0.77 (40/52) |  |  |  |  |  |  |  |  |  |  |  |  |  |  |  |  |  |  |  |  |  |  |  |  |  |  |  |  |  |  |  |  |  |  |  |  |  |  |  |  |  |  |  |  |  |  |  |  |  |  |  |  |  |  |  |  |  |
| circ1185+CA724+CA199 | 0.80 (35/44) | 0.93 (39/42) | 0.86 (74/86) | 0.92 (35/38) | 0.81 (39/48) |  |  |  |  |  |  |  |  |  |  |  |  |  |  |  |  |  |  |  |  |  |  |  |  |  |  |  |  |  |  |  |  |  |  |  |  |  |  |  |  |  |  |  |  |  |  |  |  |  |  |  |  |  |  |  |  |  |
| circ1185+CA724+CA199+CEA | 0.82 (36/44) | 0.93 (39/42) | 0.87 (75/86) | 0.92 (36/39) | 0.83 (39/47) |  |  |  |  |  |  |  |  |  |  |  |  |  |  |  |  |  |  |  |  |  |  |  |  |  |  |  |  |  |  |  |  |  |  |  |  |  |  |  |  |  |  |  |  |  |  |  |  |  |  |  |  |  |  |  |  |  |
| circ5265+CEA | 0.68 (30/44) | 0.88 (37/42) | 0.78 (67/86) | 0.86 (30/35) | 0.73 (37/51) |  |  |  |  |  |  |  |  |  |  |  |  |  |  |  |  |  |  |  |  |  |  |  |  |  |  |  |  |  |  |  |  |  |  |  |  |  |  |  |  |  |  |  |  |  |  |  |  |  |  |  |  |  |  |  |  |  |
| circ5265+CA199 | 0.93 (41/44) | 0.60 (25/42) | 0.77 (66/86) | 0.71 (41/58) | 0.89 (25/28) |  |  |  |  |  |  |  |  |  |  |  |  |  |  |  |  |  |  |  |  |  |  |  |  |  |  |  |  |  |  |  |  |  |  |  |  |  |  |  |  |  |  |  |  |  |  |  |  |  |  |  |  |  |  |  |  |  |
| circ5265+CA724 | 0.93 (41/44) | 0.60 (25/42) | 0.77 (66/86) | 0.71 (41/58) | 0.89 (25/28) |  |  |  |  |  |  |  |  |  |  |  |  |  |  |  |  |  |  |  |  |  |  |  |  |  |  |  |  |  |  |  |  |  |  |  |  |  |  |  |  |  |  |  |  |  |  |  |  |  |  |  |  |  |  |  |  |  |
| circ5265+CA724+CA199 | 0.73 (32/44) | 0.90 (38/42) | 0.81 (70/86) | 0.89 (32/36) | 0.76 (38/50) |  |  |  |  |  |  |  |  |  |  |  |  |  |  |  |  |  |  |  |  |  |  |  |  |  |  |  |  |  |  |  |  |  |  |  |  |  |  |  |  |  |  |  |  |  |  |  |  |  |  |  |  |  |  |  |  |  |
| circ5265+CA724+CA199+CEA | 0.80 (35/44) | 0.88 (38/42) | 0.84 (72/86) | 0.88 (35/40) | 0.80 (37/46) |  |  |  |  |  |  |  |  |  |  |  |  |  |  |  |  |  |  |  |  |  |  |  |  |  |  |  |  |  |  |  |  |  |  |  |  |  |  |  |  |  |  |  |  |  |  |  |  |  |  |  |  |  |  |  |  |  |
| circ1185+circ5265 | 0.66 (29/44) | 0.93 (39/42) | 0.79 (68/86) | 0.91 (29/32) | 0.72 (39/54) |  |  |  |  |  |  |  |  |  |  |  |  |  |  |  |  |  |  |  |  |  |  |  |  |  |  |  |  |  |  |  |  |  |  |  |  |  |  |  |  |  |  |  |  |  |  |  |  |  |  |  |  |  |  |  |  |  |
| circ1185+circ5265+CEA+CA199+CA724 | 0.82 (36/44) | 0.95 (40/42) | 0.88 (76/86) | 0.95 (36/38) | 0.83 (40/48) |  |  |  |  |  |  |  |  |  |  |  |  |  |  |  |  |  |  |  |  |  |  |  |  |  |  |  |  |  |  |  |  |  |  |  |  |  |  |  |  |  |  |  |  |  |  |  |  |  |  |  |  |  |  |  |  |  |

GC, gastric cancer; circ, circular; CA, carbohydrate antigen; CEA, carcinoembryonic antigen; SEN, sensitivity; SPE, specificity; ACCU, overall accuracy; NPV, negative predictive value; PPV, positive predictive value.

| **Supplementary Table 8. The diagnostic performance of circ1185, CEA, CA199, and CA724 in differentiating**  **GC patients from healthy donors in** **Affiliated Hospital of Nantong University.** | | | | | | | | | | | | | | | | | | | | | | | | | | | | | | | | | | | | | | | | | | | | | | | | | | | | | | | | | | | | | | |
| --- | --- | --- | --- | --- | --- | --- | --- | --- | --- | --- | --- | --- | --- | --- | --- | --- | --- | --- | --- | --- | --- | --- | --- | --- | --- | --- | --- | --- | --- | --- | --- | --- | --- | --- | --- | --- | --- | --- | --- | --- | --- | --- | --- | --- | --- | --- | --- | --- | --- | --- | --- | --- | --- | --- | --- | --- | --- | --- | --- | --- | --- | --- |
|  | **SEN** | **SPE** | **ACCU** | **PPV** | **NPV** |  |  |  |  |  |  |  |  |  |  |  |  |  |  |  |  |  |  |  |  |  |  |  |  |  |  |  |  |  |  |  |  |  |  |  |  |  |  |  |  |  |  |  |  |  |  |  |  |  |  |  |  |  |  |  |  |  |
| circ1185 | 0.86 (36/42) | 0.75 (30/40) | 0.80 (66/82) | 0.78 (36/46) | 0.83 (30/36) |  |  |  |  |  |  |  |  |  |  |  |  |  |  |  |  |  |  |  |  |  |  |  |  |  |  |  |  |  |  |  |  |  |  |  |  |  |  |  |  |  |  |  |  |  |  |  |  |  |  |  |  |  |  |  |  |  |
| circ5265 | 0.64 (27/42) | 0.93 (37/40) | 0.78 (64/82) | 0.90 (27/30) | 0.71 (37/52) |  |  |  |  |  |  |  |  |  |  |  |  |  |  |  |  |  |  |  |  |  |  |  |  |  |  |  |  |  |  |  |  |  |  |  |  |  |  |  |  |  |  |  |  |  |  |  |  |  |  |  |  |  |  |  |  |  |
| CEA | 0.48 (20/42) | 0.95 (38/40) | 0.71 (58/82) | 0.91 (20/22) | 0.63 (38/60) |  |  |  |  |  |  |  |  |  |  |  |  |  |  |  |  |  |  |  |  |  |  |  |  |  |  |  |  |  |  |  |  |  |  |  |  |  |  |  |  |  |  |  |  |  |  |  |  |  |  |  |  |  |  |  |  |  |
| CA199 | 0.62 (26/42) | 0.40 (16/40) | 0.51 (42/82) | 0.52 (26/50) | 0.50 (16/32) |  |  |  |  |  |  |  |  |  |  |  |  |  |  |  |  |  |  |  |  |  |  |  |  |  |  |  |  |  |  |  |  |  |  |  |  |  |  |  |  |  |  |  |  |  |  |  |  |  |  |  |  |  |  |  |  |  |
| CA724 | 0.57 (24/42) | 0.45 (18/40) | 0.51 (42/82) | 0.52 (24/46) | 0.50 (18/36) |  |  |  |  |  |  |  |  |  |  |  |  |  |  |  |  |  |  |  |  |  |  |  |  |  |  |  |  |  |  |  |  |  |  |  |  |  |  |  |  |  |  |  |  |  |  |  |  |  |  |  |  |  |  |  |  |  |
| circ1185+CEA | 0.71 (30/42) | 0.30 (12/40) | 0.51 (42/82) | 0.52 (30/58) | 0.50 (12/24) |  |  |  |  |  |  |  |  |  |  |  |  |  |  |  |  |  |  |  |  |  |  |  |  |  |  |  |  |  |  |  |  |  |  |  |  |  |  |  |  |  |  |  |  |  |  |  |  |  |  |  |  |  |  |  |  |  |
| circ1185+CA199 | 0.86 (36/42) | 0.75 (30/40) | 0.80 (66/82) | 0.78 (36/46) | 0.83 (30/36) |  |  |  |  |  |  |  |  |  |  |  |  |  |  |  |  |  |  |  |  |  |  |  |  |  |  |  |  |  |  |  |  |  |  |  |  |  |  |  |  |  |  |  |  |  |  |  |  |  |  |  |  |  |  |  |  |  |
| circ1185+CA724 | 0.83 (35/42) | 0.83 (33/40) | 0.83 (68/82) | 0.83 (35/42) | 0.83 (33/40) |  |  |  |  |  |  |  |  |  |  |  |  |  |  |  |  |  |  |  |  |  |  |  |  |  |  |  |  |  |  |  |  |  |  |  |  |  |  |  |  |  |  |  |  |  |  |  |  |  |  |  |  |  |  |  |  |  |
| circ1185+CA724+CA199 | 0.70 (29/42) | 0.98 (39/40) | 0.83 (68/82) | 0.97 (29/30) | 0.75 (39/52) |  |  |  |  |  |  |  |  |  |  |  |  |  |  |  |  |  |  |  |  |  |  |  |  |  |  |  |  |  |  |  |  |  |  |  |  |  |  |  |  |  |  |  |  |  |  |  |  |  |  |  |  |  |  |  |  |  |
| circ1185+CA724+CA199+CEA | 0.70 (29/42) | 0.98 (39/40) | 0.83 (68/82) | 0.97 (29/30) | 0.75 (39/52) |  |  |  |  |  |  |  |  |  |  |  |  |  |  |  |  |  |  |  |  |  |  |  |  |  |  |  |  |  |  |  |  |  |  |  |  |  |  |  |  |  |  |  |  |  |  |  |  |  |  |  |  |  |  |  |  |  |
| circ5265+CEA | 0.69 (29/42) | 0.95 (38/40) | 0.82 (67/82) | 0.94 (29/31) | 0.75 (38/51) |  |  |  |  |  |  |  |  |  |  |  |  |  |  |  |  |  |  |  |  |  |  |  |  |  |  |  |  |  |  |  |  |  |  |  |  |  |  |  |  |  |  |  |  |  |  |  |  |  |  |  |  |  |  |  |  |  |
| circ5265+CA199 | 0.79 (33/42) | 0.83 (33/40) | 0.80 (66/82) | 0.83 (33/40) | 0.79 (33/42) |  |  |  |  |  |  |  |  |  |  |  |  |  |  |  |  |  |  |  |  |  |  |  |  |  |  |  |  |  |  |  |  |  |  |  |  |  |  |  |  |  |  |  |  |  |  |  |  |  |  |  |  |  |  |  |  |  |
| circ5265+CA724 | 0.71 (30/42) | 0.93 (37/40) | 0.82 (67/82) | 0.91 (30/33) | 0.76 (37/49) |  |  |  |  |  |  |  |  |  |  |  |  |  |  |  |  |  |  |  |  |  |  |  |  |  |  |  |  |  |  |  |  |  |  |  |  |  |  |  |  |  |  |  |  |  |  |  |  |  |  |  |  |  |  |  |  |  |
| circ5265+CA724+CA199 | 0.83 (35/42) | 0.83 (33/40) | 0.83 (68/82) | 0.83 (35/42) | 0.83 (33/40) |  |  |  |  |  |  |  |  |  |  |  |  |  |  |  |  |  |  |  |  |  |  |  |  |  |  |  |  |  |  |  |  |  |  |  |  |  |  |  |  |  |  |  |  |  |  |  |  |  |  |  |  |  |  |  |  |  |
| circ5265+CA724+CA199+CEA | 0.71 (30/42) | 0.95 (38/40) | 0.83 (68/82) | 0.94 (30/32) | 0.76 (38/50) |  |  |  |  |  |  |  |  |  |  |  |  |  |  |  |  |  |  |  |  |  |  |  |  |  |  |  |  |  |  |  |  |  |  |  |  |  |  |  |  |  |  |  |  |  |  |  |  |  |  |  |  |  |  |  |  |  |
| circ1185+circ5265 | 0.76 (32/42) | 0.93 (37/40) | 0.84 (69/82) | 0.91 (32/35) | 0.79 (37/47) |  |  |  |  |  |  |  |  |  |  |  |  |  |  |  |  |  |  |  |  |  |  |  |  |  |  |  |  |  |  |  |  |  |  |  |  |  |  |  |  |  |  |  |  |  |  |  |  |  |  |  |  |  |  |  |  |  |
| circ1185+circ5265+CEA+CA199+CA724 | 0.74 (31/42) | 0.95 (38/40) | 0.84 (69/82) | 0.94 (31/33) | 0.78 (38/49) |  |  |  |  |  |  |  |  |  |  |  |  |  |  |  |  |  |  |  |  |  |  |  |  |  |  |  |  |  |  |  |  |  |  |  |  |  |  |  |  |  |  |  |  |  |  |  |  |  |  |  |  |  |  |  |  |  |

GC, gastric cancer; circ, circular; CA, carbohydrate antigen; CEA, carcinoembryonic antigen; SEN, sensitivity; SPE, specificity; ACCU, overall accuracy; NPV, negative predictive value; PPV, positive predictive value.
